# Exploring Phthalimide as the Acid Component in the Passerini Reaction

**DOI:** 10.1021/acs.orglett.3c03962

**Published:** 2024-01-16

**Authors:** Jingyao Li, Qiang Zheng, Alexander Dömling

**Affiliations:** † University of Groningen, Department of Drug Design, A. Deusinglaan 1, 9713 AV Groningen, The Netherlands; ‡ Institute of Molecular and Translational Medicine, Faculty of Medicine and Dentistry and Czech Advanced Technology and Research Institute, Palacký University in Olomouc, 779 00 Olomouc, Czech Republic

## Abstract

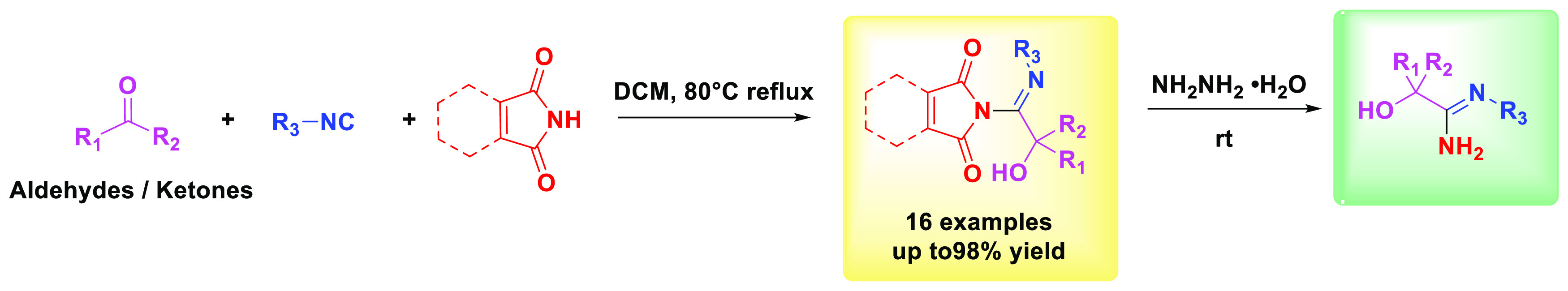

Multicomponent reactions, particularly the Passerini reaction, serve as efficient tools for the synthesis of druglike molecules and the creation of compound libraries. Despite the effectiveness of the Passerini reaction, the limited alternatives to the crucial carboxylic acid component pose a structural constraint. Here, we have discovered that the phthalimide moiety and its derivatives react in the Passerini reaction as an acid component. We explored their potential in synthesizing diverse and intricate molecules. The phthalimide moiety stands out as a favorable building block due to its oxidative stability, heat-stable characteristics, and resistance to solvents. Our approach introduces a novel perspective to multicomponent reactions by incorporating NH-based acid components, addressing the ongoing need for the development of innovative molecular scaffolds.

MCRs (multicomponent reactions) are chemical transformations that can efficiently generate a single multifunctional product by incorporating three or more reactants with almost all their atoms and are thus regarded as a considerable toolbox to expand molecular diversity and complexity in synthetic and medicinal chemistry.^1−3^ Among them, the Passerini (Figure 1a)^4^ and Ugi (Figure 1b)^5^ MCRs have experienced rapid growth with applications in diverse areas of fundamental and applied chemistry, because of the ready access of a large number of starting materials. Both reactions share a similar mechanism, which begins with the activation of the aldehyde or imine by the carboxylic acid component. However, the utilization of carboxylic acids could also be considered as a limitation of the reactions due to a finite number of readily available variations. Besides carboxylic acids, very few alternative acids were described^6,7^

**Figure 1:**
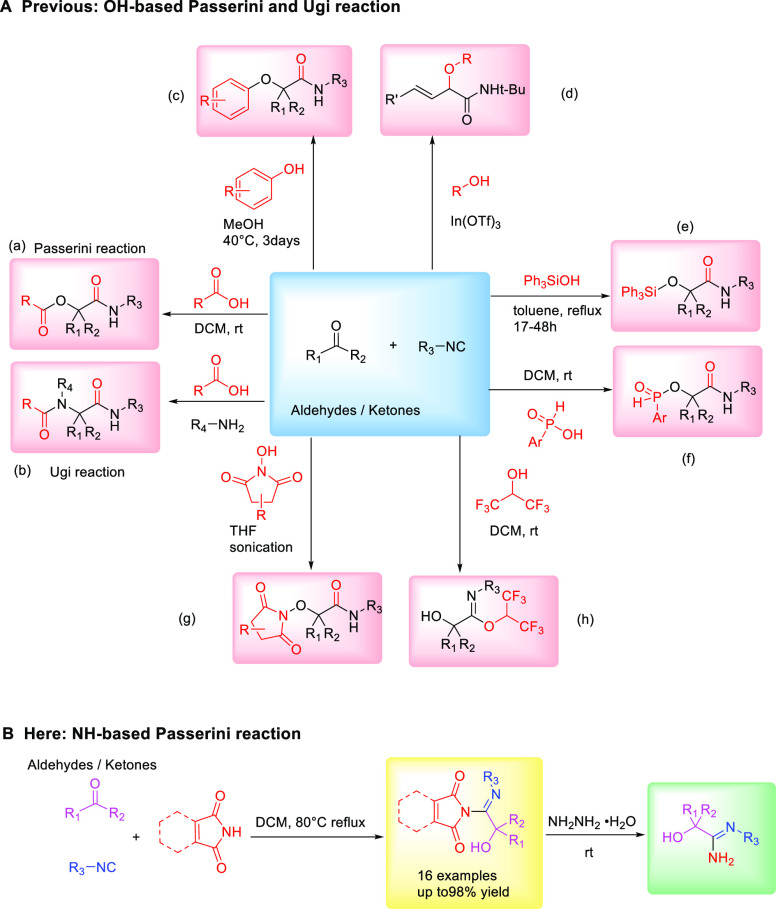
Different acid components in the Passerini reaction.

El Kaim et al. reported some notable examples by using O-arylative compounds as a replacement of carboxylic acids in both Passerini and Ugi reactions to access α-aryloxy-amides,^8^ as well as O- and N-arylamides^9^ (Figure 1c). Subsequently, the use of other starting materials instead of carboxylic acids has been reported (Figure 1A). A direct alkylative Passerini reaction was reported using free aliphatic alcohols to obtain the corresponding α-alkoxyl amide products (Figure 1d).^10^ The requirement for α,β-unsaturated aldehydes and *tert*-butyl isocyanide, in addition to the use of an In catalyst, limited the application of this methodology. Later on, O-silylative^11^ (Figure 1e) and O-phosphinative^12^ (Figure 1f) Passerini reactions were developed for the synthesis of α-siloxyamides and α-(phosphinyloxy)amides by replacing the carboxylic acid with triphenylsilanol and phenylphosphinic acid, respectively. Our group also reported N-hydroxamic acids as acid components in Passerini reaction toward α-aminoxy-amides (Figure 1g).^13^ Moreover, we published a highly improved version of the lesser-used Passerini tetrazole reaction using TMSN_3_ in water/methanol and ultrasound.^14^ In 2018, Saya et al. reported the use of hexafluoroisopropanol as an acid component in the Passerini reaction, synthesizing β-amino alcohols via a two-step one-pot approach (Figure 1h).^15^ To date, most carboxylic acid replacements in the Passerini reaction are OH-based acid bioisosteres. Thus, the discovery of non–OH-based acid components in the Passerini reaction is underexamined and of great interest.

Phthalimide, which is a bicyclic aromatic nitrogen heterocycle, has a p*K*_a_ value of 8.3 and can be considered as a carboxylic acid bioisostere. However, due to its lipophilic and neutral properties, phthalimide and its derivatives can easily cross biological membranes and therefore exhibits pharmaceutical potential.^16^ A large number of phthalimide subunits containing compounds has been designed and developed as antitumor,^17−19^ anti-inflammatory,^20,21^ anti-Alzheimer (AD),^22^ antipsychotic,^23^ antimicrobial,^24^ anticonvulsant,^25^ anxiolytic,^26^ and anti-HIV agents.^27^ Several of these compounds have reached the market for the treatment of multiple myeloma (Lenalidomide),^28^ psoriasis (Apremilast),^29^ rheumatoid arthritis, and shock septic syndrome (LASSBio-468).^30^ In addition to its abundant medicinal applications, the phthalimide moiety also plays an important role in synthetic chemistry and is considered as a precursor for the synthesis of amines^31^ and anthranilic acids.^32^

On the other hand, the acidic properties of phthalimide remain less explored. We hypothesized that the phthalimide moiety could be introduced in MCR scaffolds to form multisubstituted compounds, and, here, we explore the potential of phthalimide as the acid component in the Passerini reaction.^33^

To develop our strategy, we performed optimization of the reaction conditions. For this, isobutyraldehyde (1.0 equiv), benzyl isocyanide (1.0 equiv), and phthalimide (1.0 equiv) were utilized as starting materials (Table 1). Aprotic solvents are more preferable than protic solvents in Passerini-type reactions, and among them, DCM is the most commonly used solvent.^34^ Therefore, we started the investigation by using DCM at room temperature. However, phthalimide has poor solubility in DCM and the starting materials did not fully convert, even after 2 days of reaction, resulting in only 20% yield (Table 1, entry 1). Thus, the solubility of phthalimide is significantly hindering the reaction. We hypothesized that, by increasing the temperature of the reaction, the solubility of phthalimide could improve and, as a consequence, the reaction would be faster. Indeed, by performing the reaction at 55 °C, the yield increased to 43% (Table 1, entry 2). Moreover, the investigation of different solvents could also be a factor that could further improve the reaction. Although THF and MeOH improved the solubility of phthalimide, the yields decreased (Table 1, entries 3–5). Furthermore, the use of organic bases could form ions with the phthalimide, due to its acidic properties, and possibly help it dissolve in the organic solvents as well. However, the addition of trimethylamine failed to boost the reaction and, on the contrary, hindered the formation of product (Table 1, entries 6–9). Thus, after all the attempts, we concluded that the major factor to improve the yield of the reaction is the temperature. We returned to our initial reaction conditions and increased the temperature to 80 °C. To our delight, the increased temperature not only provided excellent yields, but also accelerated the reaction time to 4 h (Table 1, entries 10–14). Next, we tested different solvent systems with the aim of improving the solubility of phthalimide. Reactions in THF and dioxane led to lower yields (52% and 77%, respectively; see Table 1, entries 11 and 14). The use of solvent mixtures (DCM and DMF) led to good yields of 81% and 83% (Table 1, entries 12 and 13); however, pure DCM as solvent provided the best yield of 90% (Table 1, entry 10).

**Table 1:**
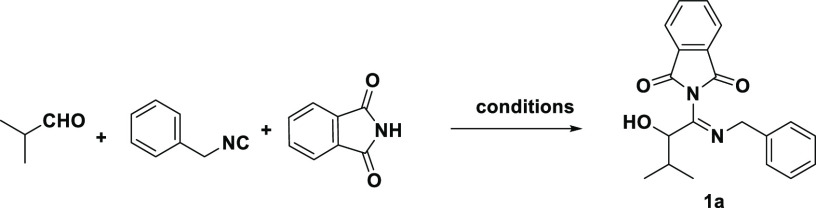
Optimization of Reaction Conditions

entry	solvent	time (h)	base	temperature (°C)	yield^a^ (%)
1	DCM	48	–	rt	20
2	DCM	12	–	55	43
3	THF	12	–	55	40
4	DCM:THF(1:1)	12	–	55	35
5	MeOH	12	–	55	trace
6	DCM	12	Et_3_N	55	ND
7	THF	12	Et_3_N	55	ND
8	DCM:DMF(9:1)	12	Et_3_N	55	ND
9	dioxane	12	Et_3_N	55	ND
10	DCM	4	–	80	90
11	THF	4	–	80	52
12	DCM:DMF(1:1)	4	–	80	81
13	DCM:DMF(9:1)	4	–	80	83
14	dioxane	4	–	80	77

a Isolated yields.

With these optimized conditions in hand, we studied the substrate scope, using diverse oxocomponents and isocyanides that were conveniently synthesized according to our recently published procedure.^35^ Initially, we used various aliphatic and aromatic isocyanides (Scheme 1). Most of the aromatic isocyanide components resulted in good to excellent yields from 74% to 99% (**1a**–**1h**). Monosubstituted aromatic isocyanides (*p*-chloro, *p*-methoxy) led to better yields of 80% and 99%, respectively, compared to nonsubstituted aromatic isocyanides (**1a**, **1b**). Additionally, 2-(2-phenyl-1*H*-indole-3-yl)ethyl isocyanide (**1d**) with a 1*H*-indole substitution led to a good yield of 78% as well. As an aliphatic isocyanide, cyclohexyl isocyanide was utilized in this reaction and resulted in a good yield, 91%. However, tert-butyl isocyanide and tert-octyl isocyanide did not give us any corresponding products. In contrast to the aromatic isocyanides, the outcome for aliphatic isocyanides depends on their substitutions.

**Scheme 1:**
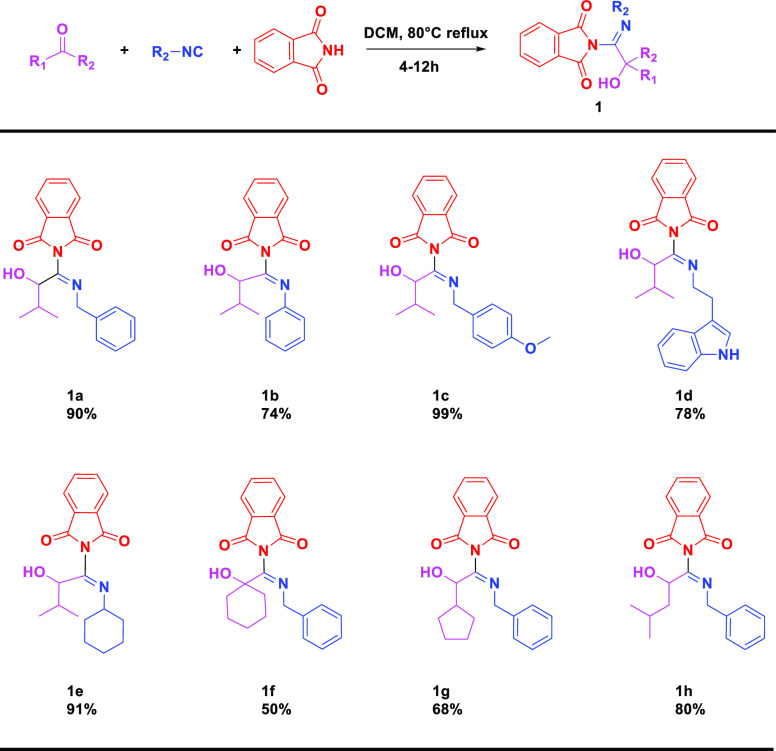
Substrate Scope of the Phthalimide Conducted Passerini Products (**1**) Isolated yield.

Next, we turned our focus to the aldehyde component. We observed that aromatic aldehydes decreased the reaction yield and only gave trace amounts of product, whereas both the linear and cyclic aliphatic aldehydes displayed moderate to good yields (80% (**1g**) and 68% (**1h**), respectively). Noteworthy, an example with a ketone was also successful, resulting in a 50% yield (**1f**). The comprehensive incorporation of different oxo and isocyanide components resulted in the creation of a compound library characterized by high complexity and diversity. This approach unveiled the notable building block availability and good functional group tolerance of the current strategy.

Besides the use of phthalimide, we envisioned that N-formylformamide-containing conjugated cyclic compounds, with low p*K*_a_ values, could be treated as acid isosteres and used in the present strategy as well (Scheme 2). The exploration started with 1*H*-pyrrole-2,5-dione (**2a**), which lacks one phenyl ring, compared with phthalimde. The lack of one phenyl ring reduced the acidic properties and led to a decreased yield of 52%, probably due to its less acidic character. On the other hand, addition of phenyl rings on different positions resulted in diverse yields. 1*H*-Benz[f]isoindole-1,3(2*H*)-dione (**2b**), which retained the basic 5-membered *N*-formylformamide cyclic scaffold of phthalimide, reacted with high yield (96%), whereas 1*H*-benzo[de]isoquinoline-1,3(2*H*)-dione (**2c**), containing a 6-membered *N*-formylformamide cyclic scaffold, resulted in only 10% yield. Furthermore, the effect of diverse substitutions was explored as well. Substituents on positions 4 and 5, including halogens (**2d**), nitro (**2e**), methyl (**2f**) and methoxyl (**2g**) substituents were well tolerated with moderate to good yields. In summary, the successful strategy using phthalimide and derivatives with different substituents in the Passerini reaction resulted in a diverse library of products.

**Scheme 2:**
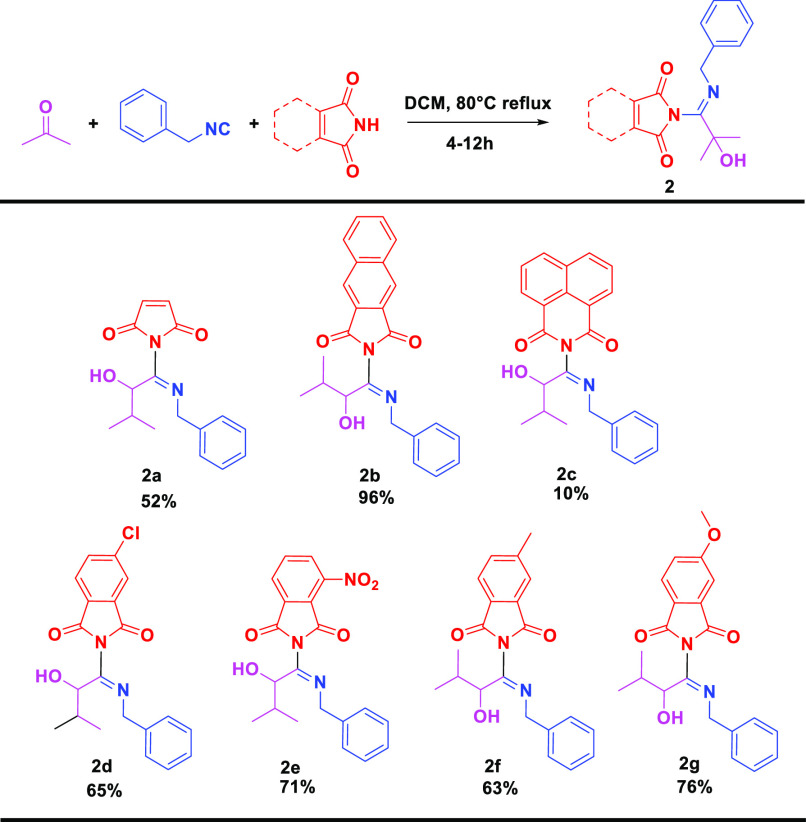
Substrate Scope of Phthalimide Derivatives (**2**) Isolated yield.

As an application of this new reaction, we next investigated the cleavage of phthalimide toward the amidine alcohols, which are important intermediates for the synthesis of bioactive heterocycles^36,37^ (Scheme 3). We first conducted the Passerine reaction in 1 mL of DCM to the formation of 2-(1-((2-(1H-indol-3-yl)ethyl)imino)-2-hydroxy-3-methylbutyl)isoindoline-1,3-dione (**1d**). The reaction was monitored by TLC and upon completion, 1 mL THF was added to the reaction mixture, followed by hydrazine. The reaction mixture was stirred for 1 h at room temperature, forming the amidine (**3a**). However, when HCl in dioxane was added to the reaction mixture, the expected HCl salt was not observed, and unexpectedly, amide (**4a**) was formed by hydrolysis. All of these reactions were conducted in a one-pot manner, without column chromatography of the intermediates (Scheme 3).

**Scheme 3:**
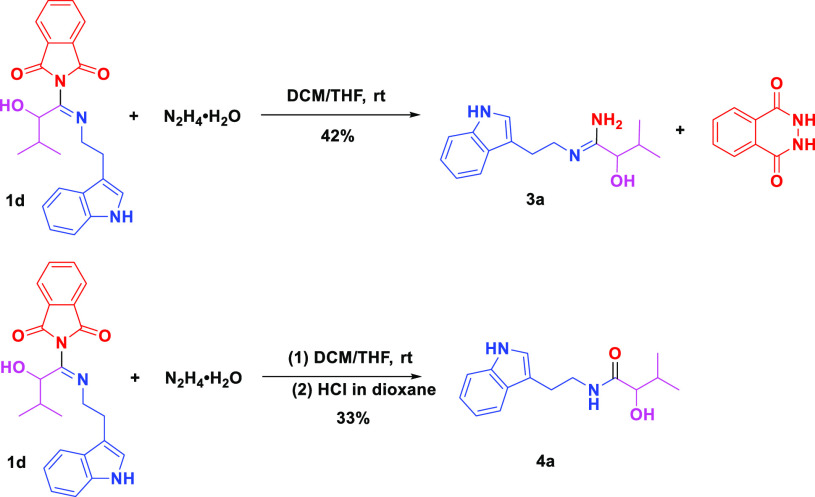
Cleavage of Phthalimide for the Synthesis of α-Amidine Alcohol (**3**) Isolated yield.

Based on the generally accepted Passerini reaction mechanism, we propose a similar mechanism (Scheme 4). Herein, the activation of the aldehyde by the acidic NH moiety of phthalimide increases the carbonyl electrophilicity, followed by the nucleophilic attack of the isocyanide to the carbonyl group of the aldehyde. Subsequently, the phtalimide −N reacts with the isocyanide carbon including the formation of a cyclic intermediate **A**. The hydrogen of phthalimide rearranges to the aldehyde oxygen and gives the corresponding alcohol.

**Scheme 4:**
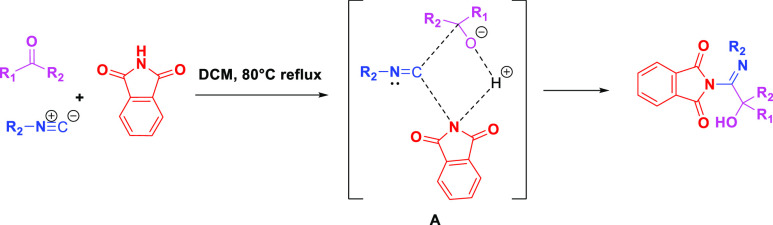
Predicted Mechanism of the Passerini Reaction

In summary, we have demonstrated an unprecedented utilization of the phthalimide moiety, serving as a substitute for the carboxylic acid component in the Passerini reaction. The methodology underwent scrutiny through a small library synthesis, showcasing a robust tolerance for both aromatic and aliphatic building blocks. The flexibility of achieving product diversity across all components was evident. The subsequent cleavage of the phthalimide facilitated the straightforward synthesis of α-amidine alcohols and α-amide alcohols, compounds typically challenging to access.^38^ Noteworthy, α-amidine alcohols are a class of compounds rarely found in organic chemistry, with an interesting hydrogen bonding pharmacophore, and are highly under-represented in medicinal chemistry.^39^ Our novel synthetic approach exhibits significant potential for applications in the screening library enrichment. Ongoing efforts in our laboratory are underway and will be reported in due course.

## Data Availability

The data underlying this study are available in the published article and its Supporting Information.

## Supporting Information Available

Experimental procedures, compound data, NMR spectra, HRMS, and crystal structure determinations (PDF)

## References

[ref1] Zarganes-Tzitzikas T.; Chandgude A. L.; Dömling A. Multicomponent reactions, union of MCRs and beyond. Chem. Rec. 2015, 15 (5), 981–996. 10.1002/tcr.201500201. 26455350

[ref2] Dömling A. Recent developments in isocyanide based multicomponent reactions in applied chemistry. Chem. Rev. 2006, 106 (1), 17–89. 10.1021/cr0505728. 16402771

[ref3] Dömling A.; Ugi I. Multicomponent reactions with isocyanides. Angew. Chem., Int. Ed. 2000, 39 (18), 3168–3210. 10.1002/1521-3773(20000915)39:18<3168::AID-ANIE3168>3.0.CO;2-U. 11028061

[ref4] Passerini M.; Simone L. Sopra gli isonitrili (I). Composto del p-isonitril-azobenzolo con acetone ed acido acetico. Gazz. Chim. Ital. 1921, 51 (II), 126–129.

[ref5] Ugi I. Versuche mit isonitrilen. Angew. Chem., Int. Ed. 1959, 71 (11), 386–386.

[ref6] El Kaim L.; Grimaud L. Beyond the Ugi reaction: less conventional interactions between isocyanides and iminium species. Tetrahedron 2009, 65 (11), 2153–2171. 10.1016/j.tet.2008.12.002.

[ref7] Hooshmand S. E.; Zhang W. Ugi Four-Component Reactions Using Alternative Reactants. Molecules 2023, 28 (4), 1642 10.3390/molecules28041642. 36838630 PMC9961709

[ref8] El Kaim L.; Gizolme M.; Grimaud L. O-arylative Passerini reactions. Org. Lett. 2006, 8 (22), 5021–5023. 10.1021/ol0617502. 17048833

[ref9] El Kaim L.; Gizolme M.; Grimaud L.; Oble J. Smiles rearrangements in Ugi-and Passerini-type couplings: New multicomponent access to O- and N-arylamides. J. Org. Chem. 2007, 72 (11), 4169–4180. 10.1021/jo070202e. 17455980

[ref10] Yanai H.; Oguchi T.; Taguchi T. Direct alkylative Passerini reaction of aldehydes, isocyanides, and free aliphatic alcohols catalyzed by indium (III) triflate. J. Org. Chem. 2009, 74 (10), 3927–3929. 10.1021/jo900354e. 19374384

[ref11] Soeta T.; Kojima Y.; Ukaji Y.; Inomata K. O-Silylative Passerini reaction: A new one-pot synthesis of α-siloxyamides. Org. Lett. 2010, 12 (19), 4341–4343. 10.1021/ol101763w. 20822154

[ref12] Soeta T.; Matsuzaki S.; Ukaji Y. A One-Pot O-Phosphinative Passerini/Pudovik Reaction: Efficient Synthesis of Highly Functionalized α-(Phosphinyloxy) amide Derivatives. Chem.–Eur. J. 2014, 20 (17), 5007–5012. 10.1002/chem.201304618. 24615915

[ref13] Chandgude A. L.; Dömling A. Unconventional Passerini Reaction toward α-Aminoxy-amides. Org. Lett. 2016, 18 (24), 6396–6399. 10.1021/acs.orglett.6b03293. 27978705

[ref14] Chandgude A. L.; Dömling A. An efficient Passerini tetrazole reaction (PT-3CR). Green Chem. 2016, 18 (13), 3718–3721. 10.1039/C6GC00910G. 27840590 PMC5102161

[ref15] Saya J. M.; Berabez R.; Broersen P.; Schuringa I.; Kruithof A.; Orru R. V.; Ruijter E. Hexafluoroisopropanol as the Acid Component in the Passerini Reaction: One-Pot Access to β-Amino Alcohols. Org. Lett. 2018, 20 (13), 3988–3991. 10.1021/acs.orglett.8b01561. 29906122 PMC6038100

[ref16] Kushwaha N.; Kaushik D. Recent advances and future prospects of phthalimide derivatives. J. Appl. Pharm. Sci. 2016, 6 (03), 159–171. 10.7324/JAPS.2016.60330.

[ref17] Singh J.; Singha T.; Naskar A.; Kundu M.; Harwansh R. K.; Mondal A.; Ghosh T.; Maity T. K. Synthesis and anti-proliferative activity of some isoindoline-1,3-dione derivatives against Ehrlich’s ascites carcinoma bearing mice model. Pharmacologyonline 2011, 2 (12), 976–987.

[ref18] Chan S. H.; Lam K. H.; Chui C. H.; Gambari R.; Yuen M. C. W.; Wong R. S. M.; Cheng G. Y. M.; Lau F. Y.; Au Y. K.; Cheng C. H. The preparation and in vitro antiproliferative activity of phthalimide based ketones on MDAMB-231 and SKHep-1 human carcinoma cell lines. Eur. J. Med. Chem. 2009, 44 (6), 2736–2740. 10.1016/j.ejmech.2008.10.024. 19081654

[ref19] Miyachi H.; Azuma A.; Ogasawara A.; Uchimura E.; Watanabe N.; Kobayashi Y.; Kato F.; Kato M.; Hashimoto Y. Novel biological response modifiers: phthalimides with tumor necrosis factor-α production-regulating activity. J. Med. Chem. 1997, 40 (18), 2858–2865. 10.1021/jm970109q. 9288167

[ref20] Alanazi A. M.; El-Azab A. S.; Al-Suwaidan I. A.; ElTahir K. E. H.; Asiri Y. A.; Abdel-Aziz N. I.; Abdel-Aziz A. A.-M. Structure-based design of phthalimide derivatives as potential cyclooxygenase-2 (COX-2) inhibitors: anti-inflammatory and analgesic activities. Eur. J. Med. Chem. 2015, 92, 115–123. 10.1016/j.ejmech.2014.12.039. 25549551

[ref21] Barbosa M. L. D.; Ramos T. J. F.; de Arantes A. C. S.; Martins M. A.; e Silva P. M. R.; Barreiro E. J.; Lima L. M. Synthesis and pharmacological evaluation of novel phenyl sulfonamide derivatives designed as modulators of pulmonary inflammatory response. Molecules 2012, 17, 14651–14672. 10.3390/molecules171214651. 23222927 PMC6268662

[ref22] Panek D.; Wieckowska A.; Wichur T.; Bajda M.; Godyn J.; Jonczyk J.; Mika K.; Janockova J.; Soukup O.; Knez D.; Korabecny J.; Gobec S.; Malawska B. Design, synthesis and biological evaluation of new phthalimide and saccharin derivatives with alicyclic amines targeting cholinesterases, beta-secretase and amyloid beta aggregation. Eur. J. Med. Chem. 2017, 125, 676–695. 10.1016/j.ejmech.2016.09.078. 27721153

[ref23] Norman M. H.; Minick D. J.; Rigdon G. C. Effect of linking bridge modifications on the antipsychotic profile of some phthalimide and isoindolinone derivatives. J. Med. Chem. 1996, 39 (1), 149–157. 10.1021/jm9502201. 8568802

[ref24] Santos J. L.; Yamasaki P. R.; Chin C. M.; Takashi C. H.; Pavan F. R.; Leite C. Q. Synthesis and in vitro anti Mycobacterium tuberculosis activity of a series of phthalimide derivatives. Bioorg. Med. Chem. 2009, 17 (11), 3795–3799. 10.1016/j.bmc.2009.04.042. 19427791

[ref25] Bhat M. A.; Al-Omar M. A. Synthesis, characterization and in vivo anticonvulsant and neurotoxicity screening of Schiff bases of phthalimide. Acta Pol. Pharm. 2011, 68 (3), 375–380. 21648191

[ref26] Hassanzadeh F.; Rabbani M.; Fasihi A.; Hakimelahi G. H.; Mohajeri M. Synthesis of phthalimide derivatives and evaluation of their anxiolytic activity. Res. Pharm. Sci. 2008, 2 (1), 35–41.

[ref27] Selvam P.; Pannecouque C.; De Clercq E. Synthesis, Anti HIV activity and Cytotoxicity of N-Substituted Phthalimide derivatives. Int. J. Pharm. Anal. Res. 2013, 2, 12–14.

[ref28] Franks M. E.; Macpherson G. R.; Figg W. D. Thalidomide. Lancet 2004, 363 (9423), 1802–1811. 10.1016/S0140-6736(04)16308-3. 15172781

[ref29] Zerilli T.; Ocheretyaner E. Apremilast (Otezla): A new oral treatment for adults with psoriasis and psoriatic arthritis. Pharm. Therapeut. 2015, 40 (8), 495. PMC4517531 26236137

[ref30] Barbosa M.; Ramos T.; de Arantes A.; Martins M.; Silva P.; Barreiro E.; Lima L. Synthesis and pharmacological evaluation of novel phenyl sulfonamide derivatives designed as modulators of pulmonary inflammatory response. Molecules 2012, 17 (12), 14651–14672. 10.3390/molecules171214651. 23222927 PMC6268662

[ref31] Gibson M. S.; Bradshaw R. W. Gabriel synthesis of primary amines. Angew. Chem. 1968, 80 (23), 986–996. 10.1002/ange.19680802303.

[ref32] Lorz P. M.; Towae F. K.; Enke W.; Jäckh R.; Bhargava N.; Hillesheim W. Phthalic acid and derivatives. In Ullmann’s Encyclopedia of Industrial Chemistry, 2000.

[ref33] Li J. Substrate Exploitation of Multicomponent Reactions toward Diverse Scaffolds and Applications in Medicinal Chemistry. Ph.D. Thesis, University of Groningen: Groningen, The Netherlands, 2021; 214 pp, 10.33612/diss.150511881.

[ref34] Banfi L.; Riva R. The P asserini Reaction. Organic reactions 2005, 65 (32), 1–140. 10.1002/0471264180.or065.01.

[ref35] Patil P.; Ahmadian-Moghaddam M.; Dömling A. Isocyanide 2.0. Green Chem. 2020, 22 (20), 6902–6911. 10.1039/D0GC02722G.

[ref36] Salimbeni A.; Canevotti R.; Paleari F.; Poma D.; Caliari S.; Fici F.; Cirillo R.; Renzetti A. R.; Subissi A. N-3-substituted pyrimidinones as potent, orally active, AT1 selective angiotensin II receptor antagonists. Journal of medicinal chemistry 1995, 38 (24), 4806–4820. 10.1021/jm00024a008. 7490730

[ref37] Xie Y.; Wang J. N-Heterocyclic carbene-catalyzed annulation of ynals with amidines: access to 1, 2, 6-trisubstituted pyrimidin-4-ones. Chem. Commun. 2018, 54 (36), 4597–4600. 10.1039/C8CC02023J. 29670951

[ref38] Tsymbalov S.; Hagen T. J.; Moore W. M.; Jerome G. M.; Connor J. R.; Manning P. T.; Pitzele B. S.; Hallinan E. A. 3-Hydroxy-4-methyl-5-pentyl-2-iminopyrrolidine: a potent and highly selective inducible nitric oxide synthase inhibitor. Bioorganic & medicinal chemistry letters 2002, 12 (22), 3337–3339. 10.1016/S0960-894X(02)00686-8. 12392746

[ref39] Haarr M. B.; Lopez O.; Fernandez-Bolanos J. G.; Lindback E.; Sydnes M. O. Functionalized d-and l-Arabino-Pyrrolidines as Potent and Selective Glycosidase Inhibitors. Synthesis 2022, 54 (12), 2916–2926. 10.1055/a-1764-8950.

