# Supplementary material for: Exploring Phthalimide as the Acid Component in the Passerini Reaction
Source: Org Lett. 2024 Jan 16;26(4):829–33. doi: 10.1021/acs.orglett.3c03962 (PMC10845143; doi:10.1021/acs.orglett.3c03962)
Supplement: Supplementary file 1 — ol3c03962_si_001.pdf [file ol3c03962_si_001.pdf]

Exploring Phthalimide as the Acid Component in the Passerini Reaction

Jingyao Li,[†§] Qiang Zheng, [†§] and Alexander Dömling[†‡*]

[†] University of Groningen, Department of Drug Design, A. Deusinglaan 1, 9713 AV Groningen, The Netherlands

[‡] Institute of Molecular and Translational Medicine, Faculty of Medicine and Dentistry and Czech Advanced Technology and Research Institute, Palacký University in Olomouc, Olomouc, Czech Republic

*e-mail: alexander.domling@upol.cz

[§]These authors contributed equally to this work.

Contents

General Information 3

Experimental Procedures...4

^{1}H NMR,^{13}C NMR and HRMS Spectral data...5

^{1}H NMR and ^{13}C NMR Spectral Graph...11

General Information

All isocyanides were made in house by performing the Ugi procedure. Other reagents were available from commercial suppliers (Sigma Aldrich, ABCR, Acros, Fluorochem and AK Scientific) and used without any purification unless otherwise noted. Thin layer chromatography was performed on Fluka precoated silica gel plates (0.20 mm thick, particle size 25 µm). Flash chromatography was performed on a Teledyne ISCO Combiflash Rf, using RediSep Rf Normal-phase Silica Flash Columns (Silica Gel 60 Å, 230 - 400 mesh) and on a Reveleris® X2 Flash Chromatography, using Grace® Reveleris Silica flash cartridges (12 grams) and a gradient of hexane/ethyl acetate (0-100%) or dichloromethane/methonal (0%-20%) was applied. Nuclear magnetic resonance spectra were recorded on a Bruker Avance 500 spectrometer. Chemical shifts for ^{1}H NMR were reported relative to TMS (δ 0 ppm) or internal solvent peak (CDCl3 δ 7.26 ppm, DMSO-d6 δ 2.50 ppm or CD3OD δ 3.31 ppm) and coupling constants were in hertz (Hz). The following abbreviations were used for spin multiplicity: s = singlet, d = doublet, t = triplet, dt = double triplet, ddd = doublet of double doublet, and m = multiplet. Chemical shifts for ^{13}C NMR reported in ppm relative to the solvent peak ($CDCl_3$ δ 77.23 ppm, MeOD δ 49.00 ppm, DMSO δ 39.52 ppm). Mass spectra were measured on a Waters Investigator Supercritical Fluid Chromatograph with a 3100 MS Detector (ESI) using a solvent system of methanol and CO_2 on a Viridis silica gel column (4.6 × 250 mm, 5 µm particle size) and reported as (m/z). High resolution mass spectra (HRMS) were recorded using a QTOF Bruker Maxis Plus, mass range 100-1500 m/z, spectra rate 2.00 Hz. Electrospray ionization mass spectra (ESI-MS) were recorded on a Waters Investigator Semi-prep 15 SFC-MS instrument. Yields given refer to chromatographically purified and spectroscopically pure compounds unless otherwise stated.

Experimental Procedures

Procedure A: General procedure for the synthesis of alcohols:

A 5 mL microwave vial equipped with a magnetic stirring bar was charged with aldehyde (1.0 mmol, 1.0 equiv), phthalimide or its derivative (1.0 mmol, 1.0 equiv) and isocyanide (1.0 mmol, 1.0 equiv) in 2mL DCM at room temperature. The vial was sealed with a cap containing a septum and subjected to metal block heating at 80 °C till completion of reaction (reaction monitored by TLC). The solvent was removed under reduced pressure and residue was purified by silica gel flash chromatography using EtOAc–hexane as eluent on to afford the titled product.

Procedure B: General procedure for the synthesis of 3a:

A 5 mL microwave vial equipped with a magnetic stirring bar was charged with an aldehyde (1.0 mmol, 1.0 equiv), phthalimide or its derivative (1.0 mmol, 1.0 equiv) and isocyanide (1.0 mmol, 1.0 equiv) in 2mL DCM at room temperature. The vial was sealed with a cap containing a septum and subjected to metal block heating at 80 °C till completion of reaction (reaction monitored by TLC). The reaction was cooled to room temperature. 2mL THF and $NH_2NH_2 \cdot H_2O$ (65μL, 1.05 mmol, 1.05 equiv) were added to the reaction, sequentially. After 1h, the reaction mixture was filtered, and the filtrate was concentrated under reduced pressure. The residue was purified by silica gel flash chromatography using DCM-MeOH as eluent on to afford the titled product.

Procedure C: General procedure for the synthesis of 4a:

A 5 ml microwave vial equipped with a magnetic stir bar was added with product 1d (195mg, 0.5 mmol, 1.0 equiv) and hydrazine hydrate (62 μL, 1.0 mmol, 2.0 equiv) in 2ml DCM/THF(1:1), the mixture was stirred under room temperature for 2h and then 0.25mL 4N HCl in dioxane (2.0 equiv) was added to the mixture. The mixture was extracted with EtOAc and then purified by silica gel flash chromatography using EtOAc-hexane as eluent to afford the product 4a.

^{1}H NMR, ^{13}C NMR and HRMS Spectral data

2-(1-(benzylimino)-2-hydroxy-3-methylbutyl)isoindoline-1,3-dione (1a)

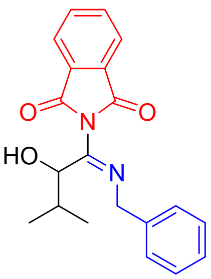

Synthesized according to procedure **A** and purified by silica gel flash chromatography using EtOAc–hexane as eluent from 1 mmol reaction as colorless liquid, yield: 303 mg (90 %); ppm. ^{1}H NMR (500 MHz, Chloroform-*d*) δ 7.91 (dd, *J* = 5.5, 3.1 Hz, 2H), 7.78 (dd, *J* = 5.5, 3.0 Hz, 2H), 7.34 – 7.26 (m, 4H), 7.25 – 7.17 (m, 1H), 4.68 (dd, *J* = 16.0, 1.8 Hz, 1H), 4.61 (dt, *J* = 3.0, 1.5 Hz, 1H), 4.50 (dd, *J* = 15.9, 1.3 Hz, 1H), 3.88 (s, 1H), 1.93 (pd, *J* = 6.8, 2.7 Hz, 1H), 1.09 (d, *J* = 6.9 Hz, 3H), 0.90 (d, *J* = 6.8 Hz, 3H). ^{13}C NMR (126 MHz, $CDCl_3$) δ 151.7, 138.2, 135.1, 131.4, 128.5, 127.8, 127.1, 124.3, 75.7, 54.8, 31.4, 20.2, 15.1. ppm HRMS (ESI-TOF) m/z: $[M + H]^+$ Calcd for $C_{20}H_{21}N_2O_3$ 337.1547; Found 337.1545.

2-(2-hydroxy-3-methyl-1-(phenylimino)butyl)isoindoline-1,3-dione (1b)

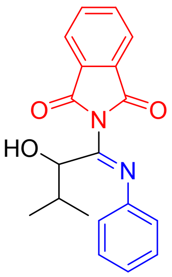

Synthesized according to procedure **A** and purified by silica gel flash chromatography using EtOAc–hexane as eluent from 1 mmol reaction as colorless liquid, yield: 239 mg (74 %); ppm. ^{1}H NMR (500 MHz, Chloroform-*d*) δ 7.83 – 7.75 (m, 2H), 7.74 – 7.66 (m, 2H), 7.24 – 7.13 (m, 2H), 7.05 – 6.95 (m, 1H), 6.88 – 6.80 (m, 2H), 4.68 (d, *J* = 2.9 Hz, 1H), 3.75 (s, 1H), 2.14 – 1.96 (m, 1H), 1.15 (d, *J* = 6.9 Hz, 3H), 1.02 (d, *J* = 6.8 Hz, 3H). ^{13}C NMR (126 MHz, $CDCl_3$) δ 152.3, 146.5, 134.8, 131.1, 128.9, 125.1, 124.1, 119.4, 76.3, 31.2, 20.2, 15.1. ppm HRMS (ESI-TOF) m/z: $[M + H]^+$ Calcd for $C_{19}H_{19}N_2O_3$ 323.1390; Found 323.1389.

2-(2-hydroxy-1-((4-methoxybenzyl)imino)-3-methylbutyl)isoindoline-1,3-dione (1c)

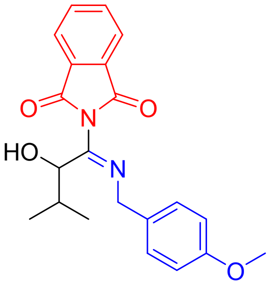

Synthesized according to procedure **A** and purified by silica gel flash chromatography using EtOAc–hexane as eluent from 1 mmol reaction as colorless liquid, yield: 363 mg (99 %); ppm. ^{1}H NMR (500 MHz, Chloroform-*d*) δ 7.91 (dd, *J* = 5.5, 3.0 Hz, 2H), 7.80 (dd, *J* = 5.5, 3.1 Hz, 2H), 7.21 (d, *J* = 8.6 Hz, 2H), 6.83 (d, *J* = 8.7 Hz, 2H), 4.67 – 4.55 (m, 2H), 4.45 (d, *J* = 15.4 Hz, 1H), 3.75 (s, 3H), 1.92 (pd, *J* = 6.8, 2.6 Hz, 1H), 1.08 (d, *J* = 7.0 Hz, 3H), 0.90 (d, *J* = 6.8 Hz, 3H). ^{13}C NMR (126 MHz, $CDCl_3$) δ 158.7, 151.2, 135.0, 131.4, 130.3, 128.9, 124.3, 113.9, 75.6, 55.2, 54.3, 31.3, 20.1, 15.1. ppm HRMS (ESI-TOF) m/z: $[M + H]^+$ Calcd for $C_{21}H_{23}N_2O_4$ 367.1652; Found 367.1652.

2-(1-((2-(1H-indol-3-yl)ethyl)imino)-2-hydroxy-3-methylbutyl)isoindoline-1,3-dione (1d)

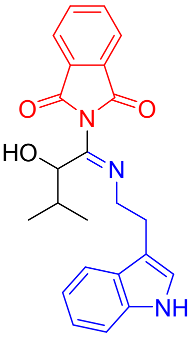

Synthesized according to procedure **A** and purified by silica gel flash chromatography using EtOAc–hexane as eluent from 1 mmol reaction as colorless liquid, yield: 304 mg (78 %); ppm. ^{1}H NMR (500 MHz, Chloroform-*d*) δ 8.45 – 8.33 (m, 1H), 7.85 – 7.69 (m, 2H), 7.68 – 7.61 (m, 2H), 7.45 (d, *J* = 8.0 Hz, 1H), 7.25 (d, *J* = 8.2 Hz, 1H), 7.04 (ddd, *J* = 8.1, 6.9, 1.1 Hz, 1H),

6.93 (d, J = 2.3 Hz, 1H), 6.87 (ddd, J = 8.0, 7.0, 1.0 Hz, 1H), 4.56 – 4.49 (m, 1H), 3.86 – 3.76 (m, 1H), 3.70 – 3.60 (m, 1H), 3.23 – 3.12 (m, 1H), 3.11 – 3.01 (m, 1H), 1.85 (pd, J = 6.8, 2.7 Hz, 1H), 1.06 (d, J = 6.9 Hz, 3H), 0.87 (d, J = 6.8 Hz, 3H). ^{13}C NMR (126 MHz, $CDCl_3$) δ 150.6, 136.4, 134.8, 131.2, 127.3, 124.1, 122.6, 121.8, 119.1, 118.5, 113.1, 111.3, 75.5, 51.8, 31.3, 26.3, 20.2, 15.1. ppm HRMS (ESI-TOF) m/z: $[M + H]^+$ Calcd for $C_{23}H_{24}N_3O_3$ 390.1812; Found 390.1811.

2-(1-(cyclohexylimino)-2-hydroxy-3-methylbutyl)isoindoline-1,3-dione (**1e**)

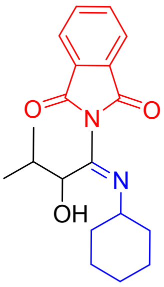

Synthesized according to procedure **A** and purified by silica gel flash chromatography using EtOAc–hexane as eluent from 1 mmol reaction as colorless liquid, yield: 299 mg (91 %); ppm. ^{1}H NMR (500 MHz, Chloroform-d) δ 7.96 (dd, J = 5.5, 3.1 Hz, 2H), 7.87 (dd, J = 5.6, 3.1 Hz, 2H), 4.38 (d, J = 2.6 Hz, 1H), 3.94 (s, 1H), 3.43 – 3.16 (m, 1H), 1.91 – 1.82 (m, 2H), 1.82 – 1.68 (m, 2H), 1.65 – 1.46 (m, 4H), 1.30 – 1.22 (m, 3H), 1.06 (d, J = 6.9 Hz, 3H), 0.87 (d, J = 6.8 Hz, 3H). ^{13}C NMR (126 MHz, $CDCl_3$) δ 147.8, 134.8, 131.4, 124.1, 75.5, 59.5, 33.5, 32.9, 30.7, 25.5, 24.0, 23.9, 20.1, 14.7. ppm HRMS (ESI-TOF) m/z: $[M + H]^+$ Calcd for $C_{19}H_{25}N_2O_3$ 329.1860; Found 329.1862.

2-((benzylimino)(1-hydroxycyclohexyl)methyl)isoindoline-1,3-dione (**1f**)

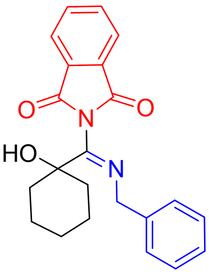

Synthesized according to procedure **A** and purified by silica gel flash chromatography using EtOAc–hexane as eluent from 1 mmol reaction as colorless liquid, yield: 181 mg (50 %); ppm. ^{1}H NMR (500 MHz, Chloroform-d) δ 7.92 (dd, J = 5.5, 3.1 Hz, 2H), 7.80 (dd, J = 5.5, 3.0 Hz, 2H), 7.36 – 7.27 (m, 4H), 7.26 – 7.20 (m, 1H), 4.48 (s, 2H), 1.89 – 1.79 (m, 4H), 1.75 – 1.58 (m, 6H). ^{13}C NMR (126 MHz, $CDCl_3$) δ 166.5, 155.5, 137.9, 134.7, 134.3, 131.6, 128.4, 127.7, 127.1, 124.1, 123.6, 75.8, 55.0, 35.5, 25.3, 21.4. ppm HRMS (ESI-TOF) m/z: $[M + H]^+$ Calcd for $C_{22}H_{23}N_2O_3$ 363.1703; Found 363.1696.

2-(1-(benzylimino)-2-cyclopentyl-2-hydroxyethyl)isoindoline-1,3-dione (**1g**)

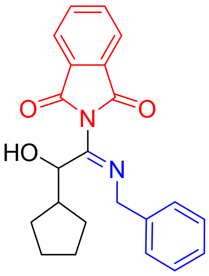

Synthesized according to procedure **A** and purified by silica gel flash chromatography using EtOAc–hexane as eluent from 1 mmol reaction as colorless liquid, yield: 247 mg (68 %); ^{1}H NMR (500 MHz, Chloroform-d) δ 7.95 (dd, J = 5.5, 3.1 Hz, 2H), 7.83 (dd, J = 5.5, 3.0 Hz, 2H), 7.36 – 7.30 (m, 4H), 7.28 – 7.23 (m, 1H), 4.75 – 4.71 (m, 1H), 4.68 (dd, J = 15.9, 1.7 Hz, 1H), 4.53 (dd, J = 15.9, 1.2 Hz, 1H), 4.15 – 3.68 (m, 1H), 2.21 – 2.09 (m, 1H), 1.80 – 1.70 (m, 1H), 1.70 – 1.59 (m, 4H), 1.57 – 1.43 (m, 3H). ^{13}C NMR (126 MHz,

CDCl3) δ 151.9, 138.2, 135.1, 131.4, 128.5, 127.8, 127.1, 124.3, 73.7, 54.7, 42.7, 29.5, 26.0, 25.9. ppm. HRMS (ESI-TOF) m/z: $[M + H]^+$ Calcd for $C_{22}H_{23}N_2O_3$ 363.1703; Found 363.1691.

2-(1-(benzylimino)-2-hydroxy-4-methylpentyl)isoindoline-1,3-dione (**1h**)

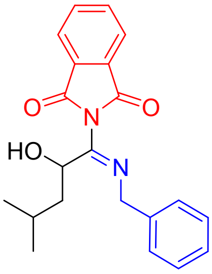

Synthesized according to procedure **A** and purified by silica gel flash chromatography using EtOAc–hexane as eluent from 1 mmol reaction as colorless liquid, yield: 280 mg (80 %); ^{1}H NMR (500 MHz, Chloroform-*d*) δ 7.93 (dd, *J* = 5.5, 3.1 Hz, 2H), 7.80 (dd, *J* = 5.5, 3.0 Hz, 2H), 7.34 – 7.27 (m, 4H), 7.26 – 7.20 (m, 1H), 4.75 – 4.68 (m, 1H), 4.62 (dd, *J* = 15.9, 1.7 Hz, 1H), 4.50 (dd, *J* = 15.8, 1.3 Hz, 1H), 2.06 – 1.89 (m, 1H), 1.63 – 1.55 (m, 1H), 1.46 – 1.35 (m, 1H), 0.92 (s, 3H), 0.91 (s, 3H). ^{13}C NMR (126 MHz, $CDCl_3$) δ 152.3, 138.1, 135.0, 131.4, 128.5, 127.8, 127.2, 124.3, 70.5, 54.9, 43.5, 24.7, 23.7, 21.4. ppm HRMS (ESI-TOF) m/z: $[M + H]^+$ Calcd for $C_{21}H_{23}N_2O_3$ 351.1703; Found 351.1704.

1-(1-(benzylimino)-2-hydroxy-3-methylbutyl)-1*H*-pyrrole-2,5-dione (**2a**)

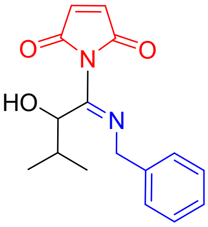

Synthesized according to procedure **A** and purified by silica gel flash chromatography using EtOAc–hexane as eluent from 1 mmol reaction as colorless liquid, yield: 149 mg (52 %);^{1}H NMR (500 MHz, Chloroform-*d*) δ 7.37 – 7.31 (m, 2H), 7.30 – 7.24 (m, 3H), 6.81 (s, 2H), 4.61 (dd, *J* = 16.0, 1.7 Hz, 1H), 4.52 – 4.46 (m, 1H), 4.42 (dd, *J* = 16.0, 1.3 Hz, 1H), 3.88 – 3.72 (m, 1H), 1.87 – 1.75 (m, 1H), 1.07 (d, *J* = 6.9 Hz, 3H), 0.84 (d, *J* = 6.8 Hz, 3H). ^{13}C NMR (126 MHz, $CDCl_3$) δ 150.6, 138.0, 134.8, 128.5, 127.7, 127.2, 75.3, 54.6, 31.5, 20.0, 15.0. ppm. HRMS (ESI-TOF) m/z: $[M + H]^+$ Calcd for $C_{16}H_{19}N_2O_3$ 287.1390; Found 287.1391.

2-(1-(benzylimino)-2-hydroxy-3-methylbutyl)-1*H*-benzo[f]isoindole-1,3(2*H*)-dione (**2b**)

Synthesized according to procedure **A** and purified by silica gel flash chromatography using

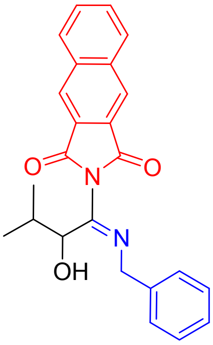

EtOAc–hexane as eluent from 1 mmol reaction as colorless liquid, yield: 371 mg (96 %); ^{1}H NMR (500 MHz, Chloroform-*d*) δ 8.39 (s, 2H), 8.03 (dd, *J* = 6.2, 3.3 Hz, 2H), 7.70 (dd, *J* = 6.3, 3.3 Hz, 2H), 7.35 – 7.11 (m, 6H), 4.76 – 4.65 (m, 2H), 4.56 (dd, *J* = 16.4, 1.5 Hz, 1H), 4.03 (s, 1H), 2.12 – 1.91 (m, 1H), 1.12 (d, *J* = 6.9 Hz, 3H), 0.97 (d, *J* = 6.8 Hz, 3H). ^{13}C NMR (126 MHz, $CDCl_3$) δ 152.1, 138.3, 135.7, 130.5, 129.9, 128.5, 127.8, 127.1, 126.7, 126.2, 75.8, 54.9, 31.5, 20.2, 15.3. ppm HRMS (ESI-TOF) m/z: $[M + H]^+$ Calcd for $C_{24}H_{23}N_2O_3$ 387.1702; Found 387.1702.

2-(1-(benzylimino)-2-hydroxy-3-methylbutyl)-1*H*-benzo[de]isoquinoline-1,3(2*H*)-dione (2c)

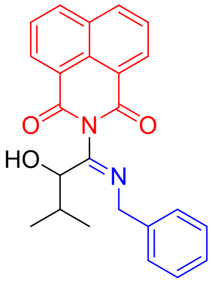

Synthesized according to procedure **A** and purified by silica gel flash chromatography using EtOAc–hexane as eluent from 1 mmol reaction as colorless liquid, yield: 38 mg (10 %); ^{1}H NMR (500 MHz, Chloroform-*d*) δ 8.64 (td, *J* = 7.4, 1.1 Hz, 2H), 8.30 (dt, *J* = 8.2, 1.2 Hz, 2H), 7.81 (ddd, *J* = 8.6, 7.4, 1.8 Hz, 2H), 7.37 – 7.18 (m, 5H), 4.59 (dd, *J* = 15.9, 1.5 Hz, 1H), 4.50 (dd, *J* = 15.9, 1.3 Hz, 1H), 4.47 – 4.42 (m, 1H), 3.62 (s, 1H), 2.10 – 1.98 (m, 1H), 1.10 (d, *J* = 6.9 Hz, 3H), 1.03 (d, *J* = 6.7 Hz, 3H). ^{13}C NMR (126 MHz, $CDCl_3$) δ 163.1, 162.4, 153.8, 138.2, 135.0, 132.0, 131.9, 131.8, 128.8, 128.4, 127.9, 127.2, 127.0, 122.0, 121.8, 77.3, 54.4, 30.9, 20.6, 15.2.ppm. HRMS (ESI-TOF) m/z: $[M + H]^+$ Calcd for $C_{24}H_{23}N_2O_3$ 387.1703; Found 387.1703.

2-(1-(benzylimino)-2-hydroxy-3-methylbutyl)-5-chloroisoindoline-1,3-dione (2d)

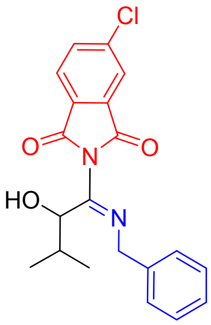

Synthesized according to procedure **A** and purified by silica gel flash chromatography using EtOAc–hexane as eluent from 1 mmol reaction as colorless liquid, yield: 240 mg (65 %); ^{1}H NMR (500 MHz, Chloroform-*d*) δ 7.97 – 7.81 (m, 2H), 7.74 (dd, *J* = 8.0, 1.8 Hz, 1H), 7.33 – 7.26 (m, 4H), 7.24 – 7.20 (m, 1H), 4.66 (dd, *J* = 16.1, 1.8 Hz, 1H), 4.62 – 4.54 (m, 1H), 4.48 (dd, *J* = 16.0, 1.3 Hz, 1H), 3.84 (s, 1H), 1.97 – 1.80 (m, 1H), 1.08 (d, *J* = 6.9 Hz, 3H), 0.88 (d, *J* = 6.8 Hz, 3H).ppm. HRMS (ESI-TOF) m/z: $[M + H]^+$ Calcd for $C_{20}H_{20}ClN_2O_3$ 371.1157; Found 371.1162.

2-(1-(benzylimino)-2-hydroxy-3-methylbutyl)-4-nitroisoindoline-1,3-dione (2e)

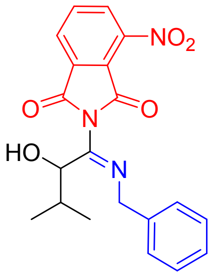

Synthesized according to procedure **A** and purified by silica gel flash chromatography using EtOAc–hexane as eluent from 1 mmol reaction as colorless liquid, yield: 271 mg (71 %); ^{1}H NMR (500 MHz, Chloroform-*d*) δ 8.25 – 8.14 (m, 2H), 8.01 (t, *J* = 7.9 Hz, 1H), 7.36 – 7.27 (m, 5H), 7.26 – 7.18 (m, 1H), 4.70 (d, *J* = 16.0 Hz, 1H), 4.59 – 4.49 (m, 2H), 2.02 – 1.90 (m, 1H), 1.10 (d, *J* = 6.9 Hz, 3H), 0.91 (d, *J* = 6.8 Hz, 3H). ^{13}C NMR (126 MHz, $CDCl_3$) δ 150.4, 145.4, 137.7, 136.6, 133.2, 129.6, 128.7, 128.6, 128.1, 127.7, 127.3, 123.0, 75.8, 54.9, 31.4, 20.1, 15.1.ppm. HRMS (ESI-TOF) m/z: $[M + H]^+$ Calcd for $C_{20}H_{20}N_3O_5$ 382.1397; Found 382.1397.

2-(1-(benzylimino)-2-hydroxy-3-methylbutyl)-5-methylisoindoline-1,3-dione (2f)

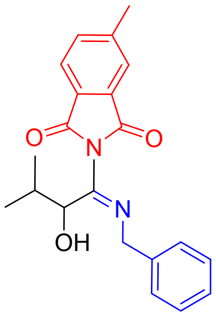

Synthesized according to procedure **A** and purified by silica gel flash chromatography using EtOAc–hexane as eluent from 1 mmol reaction as colorless liquid, yield: 221 mg (63 %); ^{1}H NMR (500 MHz, Chloroform-*d*) δ 7.83 (d, *J* = 7.7 Hz, 1H), 7.75 (s, 1H), 7.62 (dt, *J* = 7.7, 1.1 Hz, 1H), 7.37 – 7.30 (m, 4H), 7.30 – 7.22 (m, 1H), 4.71 (dd, *J* = 16.0, 1.8 Hz, 1H), 4.65 (dt, *J* = 3.1, 1.6 Hz, 1H), 4.53 (dd, *J* = 16.0, 1.3 Hz, 1H), 3.95 (s, 1H), 2.56 (s, 3H), 1.99 – 1.87 (m, 1H), 1.12 (d, *J* = 6.9 Hz, 3H), 0.92 (d, *J* = 6.8 Hz, 3H). ^{13}C NMR (126 MHz, $CDCl_3$) δ 151.8, 146.7, 138.3, 135.7, 131.8, 128.8, 128.5, 127.7, 127.1, 124.8, 124.2, 75.5, 54.8, 31.4, 22.1, 20.1, 15.1.ppm. HRMS (ESI-TOF) m/z: $[M + H]^+$ Calcd for $C_{21}H_{23}N_2O_3$ 351.1703; Found 351.1702.

2-(1-(benzylimino)-2-hydroxy-3-methylbutyl)-5-methoxyisoindoline-1,3-dione (**2g**)

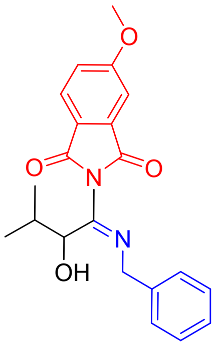

Synthesized according to procedure **A** and purified by silica gel flash chromatography using EtOAc–hexane as eluent from 1 mmol reaction as colorless liquid, yield: 278 mg (76 %); ^{1}H NMR (500 MHz, Chloroform-*d*) δ 7.81 (d, *J* = 8.4 Hz, 1H), 7.37 (d, *J* = 2.4 Hz, 1H), 7.34 – 7.27 (m, 4H), 7.26 – 7.18 (m, 2H), 4.67 (dd, *J* = 16.1, 1.8 Hz, 1H), 4.61 (dt, *J* = 3.0, 1.6 Hz, 1H), 4.50 (dd, *J* = 16.0, 1.3 Hz, 1H), 3.91 (s, 3H), 1.96 – 1.86 (m, 1H), 1.08 (d, *J* = 6.9 Hz, 3H), 0.89 (d, *J* = 6.8 Hz, 3H). ^{13}C NMR (126 MHz, $CDCl_3$) δ 165.4, 151.8, 138.3, 134.0, 128.7, 128.5, 127.7, 127.1, 126.1, 123.1, 121.1, 108.8, 75.5, 56.3, 54.8, 31.4, 20.1, 15.1.ppm. HRMS (ESI-TOF) m/z: $[M + H]^+$ Calcd for $C_{21}H_{23}N_2O_4$ 367.1652; Found 367.1652.

(*Z*)-*N'*-(2-(1*H*-indol-3-yl)ethyl)-2-hydroxy-3-methylbutanimidamide(3a)

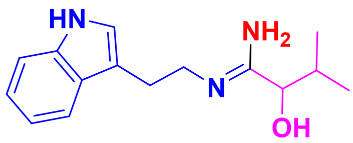

Synthesized according to procedure **B** and purified by silica gel flash chromatography using DCM-MeOH as eluent from 1 mmol reaction as colorless liquid, yield: 109 mg (42 %);^{1}H NMR (500 MHz, Methanol-d_4) δ 7.59 (d, *J* = 7.9 Hz, 1H), 7.36 (d, *J* = 8.1 Hz, 1H), 7.16 (s, 1H), 7.13 (t, *J* = 7.6 Hz, 1H), 7.05 (t, *J* = 7.4 Hz, 1H), 4.12 (d, *J* = 3.9 Hz, 1H), 3.65 (t, *J* = 7.0 Hz, 2H), 3.12 (t, *J* = 7.1 Hz, 2H), 1.91 – 1.84 (m, 1H), 0.99 (d, *J* = 6.9 Hz, 3H), 0.78 (d, *J* = 6.8 Hz, 3H). ^{13}C NMR (126 MHz, MeOD) δ 158.5, 132.0, 131.7, 128.6, 126.9, 125.4, 110.1, 42.3, 32.9, 23.4, 14.1. ppm. HRMS (ESI-TOF) m/z: $[M + H]^+$ Calcd for $C_{15}H_{22}N_3O$ 260.1757; Found 260.1756.

N-(2-(1H-indol-3-yl)ethyl)-2-hydroxy-3-methylbutanamide(4a)

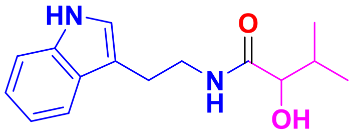

Synthesized according to procedure **C** and purified by silica gel flash chromatography using EtOAc–hexane as eluent from 1 mmol reaction as colorless liquid, yield: 86 mg (33 %); ^{1}H NMR (500 MHz, Methanol-d_4) δ 8.12 (dd, J = 5.9, 3.4 Hz, 1H), 7.68 (dd, J = 6.0, 3.3 Hz, 1H), 7.47 (d, J = 7.9 Hz, 1H), 7.26 (d, J = 8.1 Hz, 1H), 7.01 (t, J = 7.5 Hz, 1H), 6.93 (t, J = 7.4 Hz, 1H), 4.05 (d, J = 4.0 Hz, 1H), 3.55 (t, J = 7.1 Hz, 2H), 3.01 (t, J = 7.1 Hz, 2H), 1.81 – 1.74 (m, 1H), 1.23 (s, 1H), 0.86 (d, J = 6.8 Hz, 3H), 0.66 (d, J = 6.8 Hz, 3H). ^{13}C NMR (126 MHz, MeOD) δ 169.4, 136.9, 127.0, 122.7, 121.2, 118.5, 111.0, 110.1, 105.9, 42.3, 32.8, 23.4, 18.0, 14.1. ppm HRMS (ESI-TOF) m/z: $[M + H]^+$ Calcd for $C_{15}H_{21}N_2O_2$ 261.1598; Found 261.1590.

^{1}H NMR and ^{13}C NMR Spectral Graph

2-(1-(benzylimino)-2-hydroxy-3-methylbutyl)isoindoline-1,3-dione (1a)

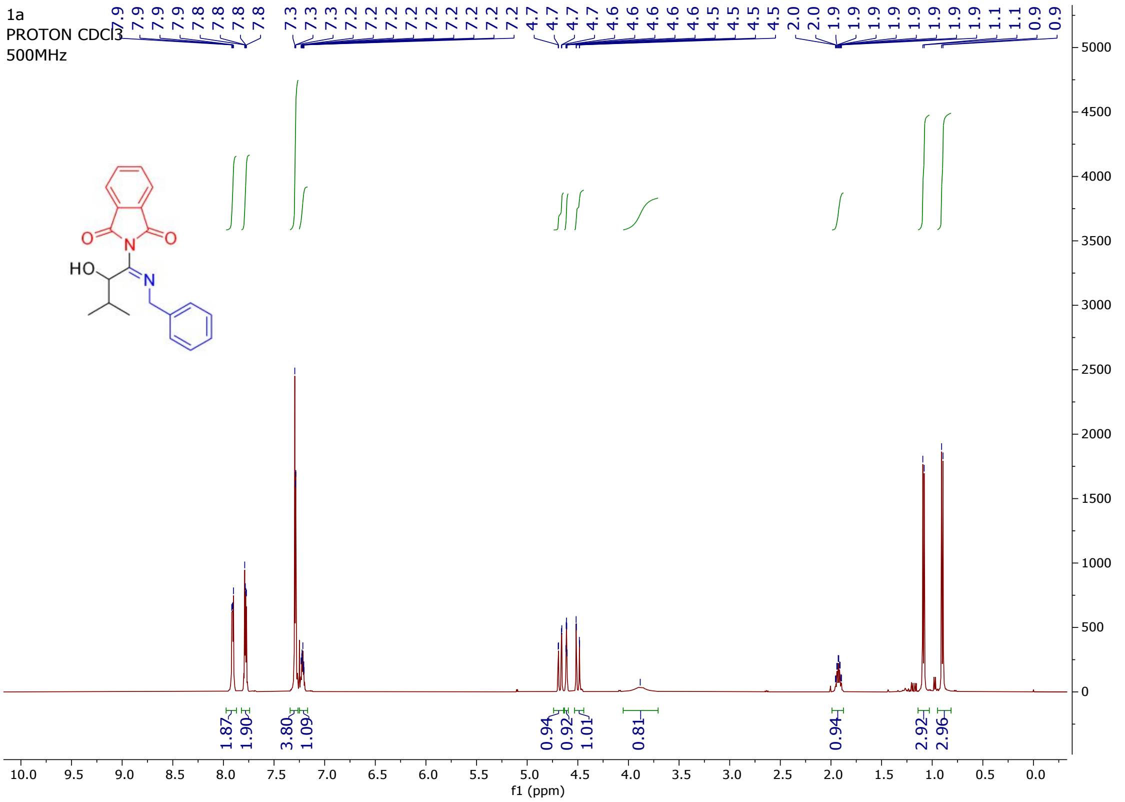

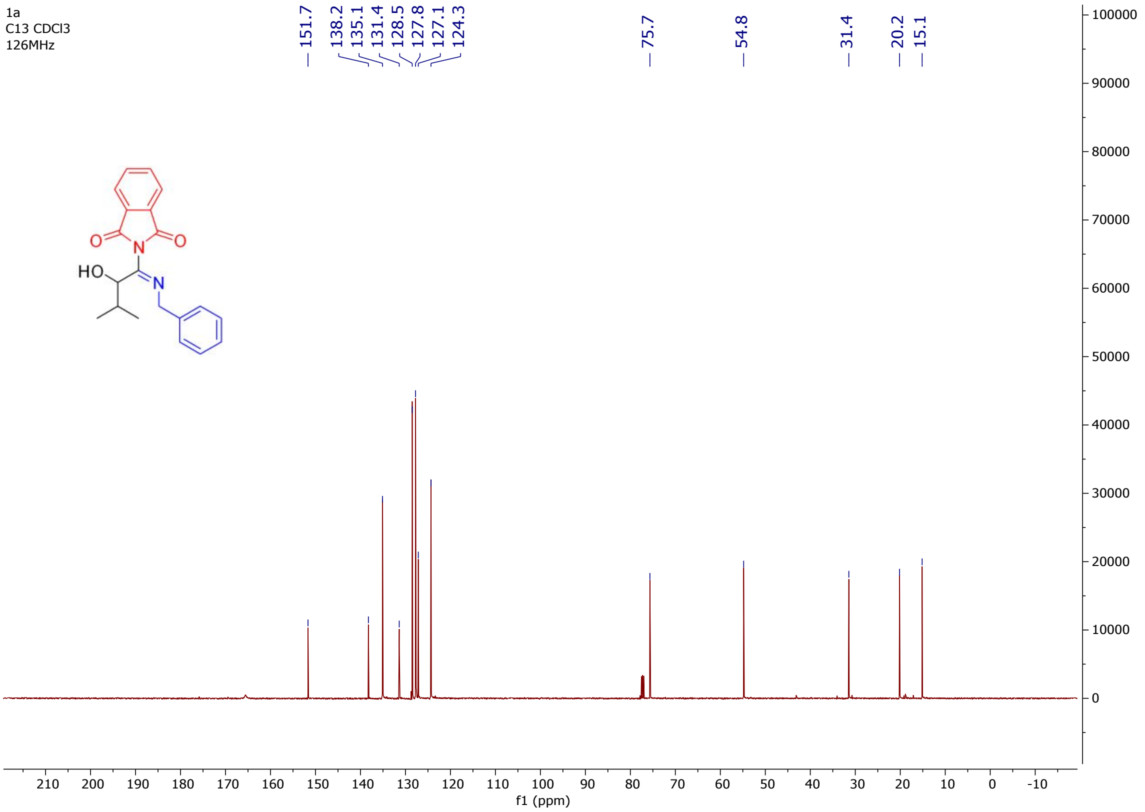

2-(2-hydroxy-3-methyl-1-(phenylimino)butyl)isoindoline-1,3-dione (1b)

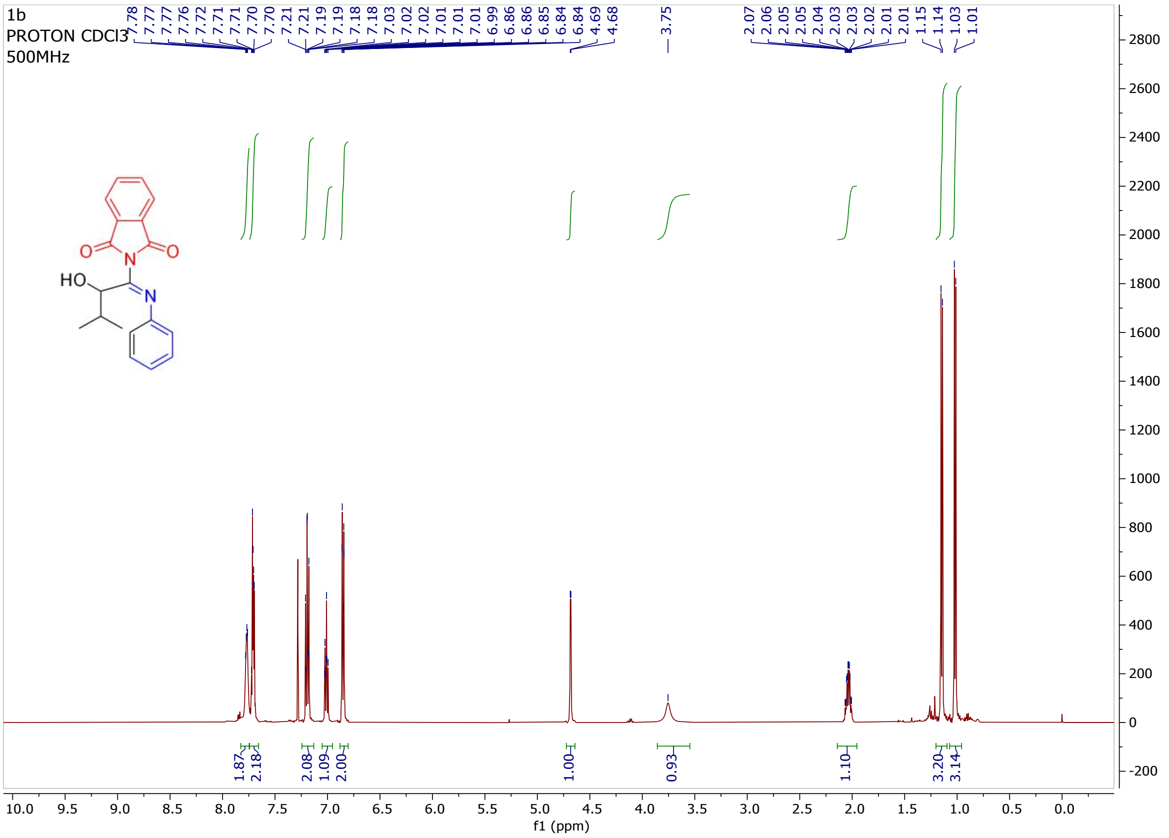

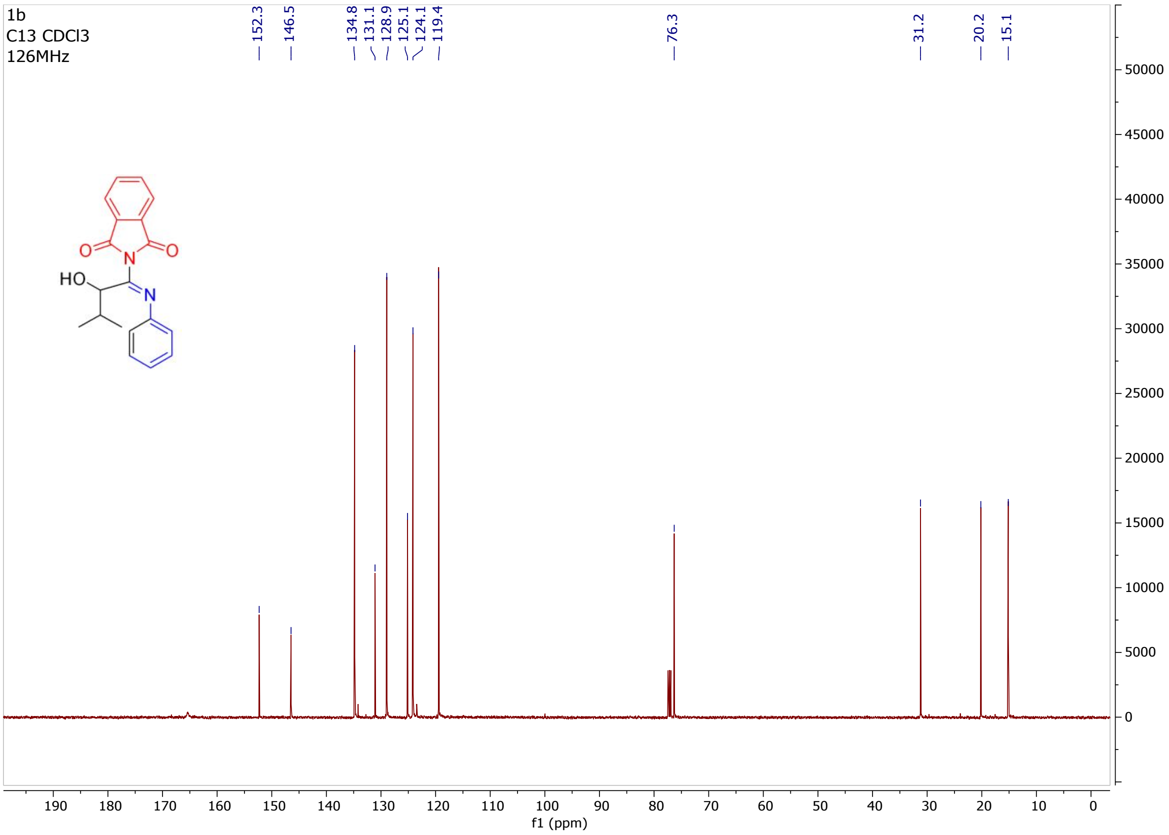

2-(2-hydroxy-1-((4-methoxybenzyl)imino)-3-methylbutyl)isoindoline-1,3-dione (1c)

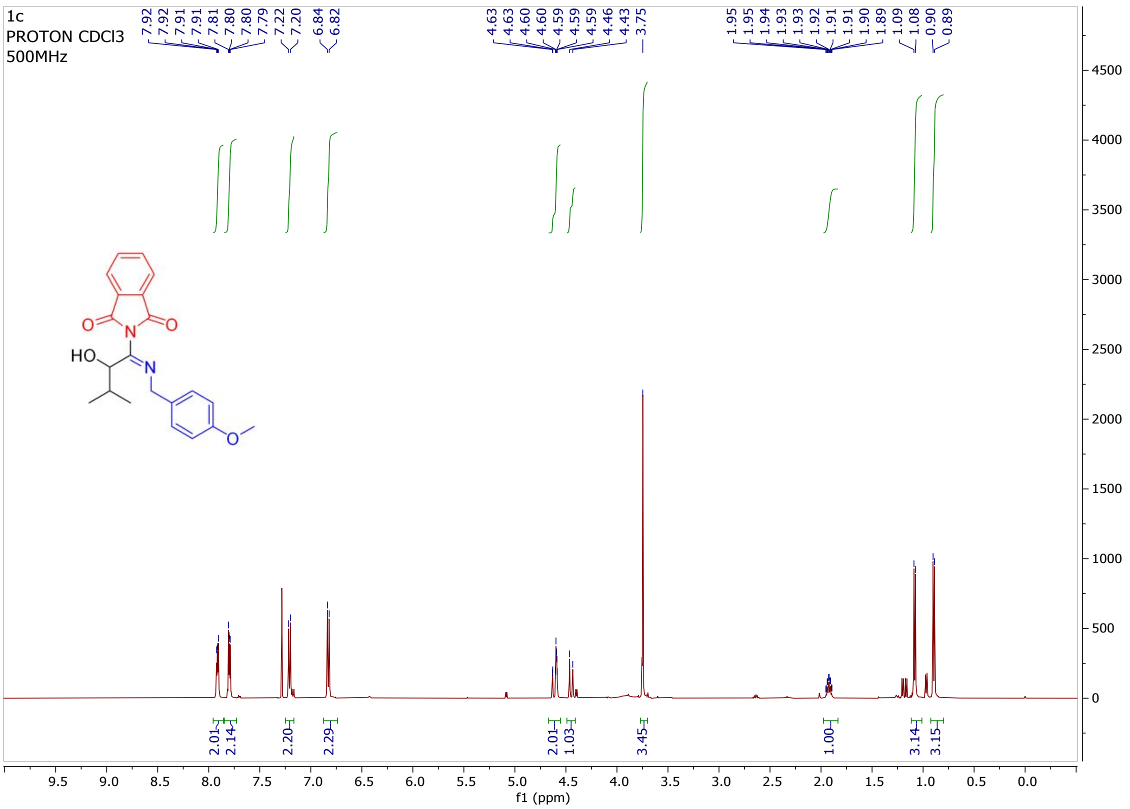

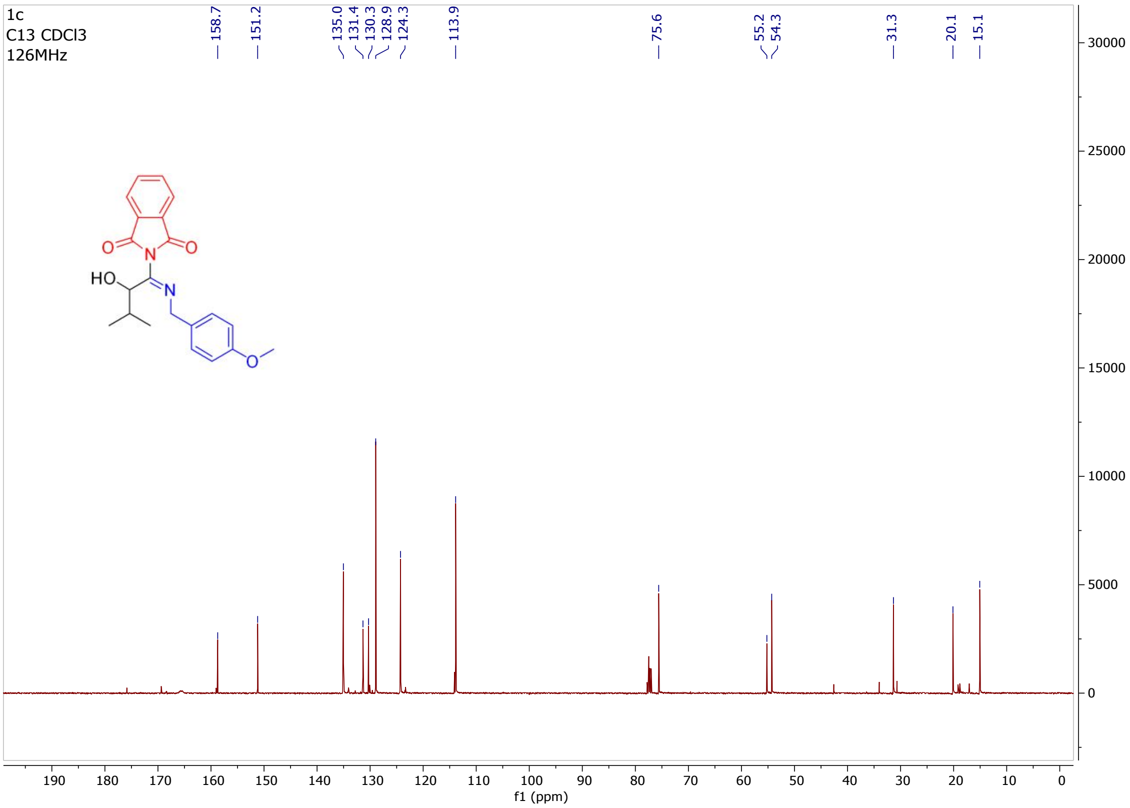

2-(1-((2-(1*H*-indol-3-yl)ethyl)imino)-2-hydroxy-3-methylbutyl)isoindoline-1,3-dione (1d)

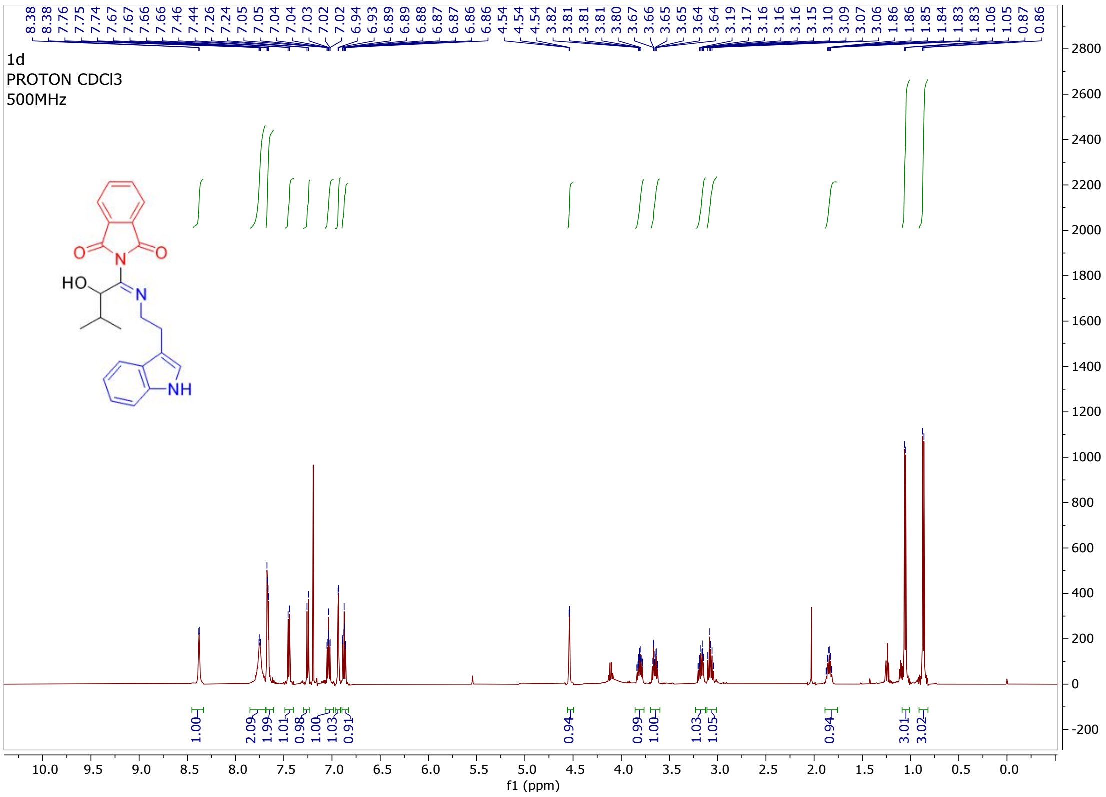

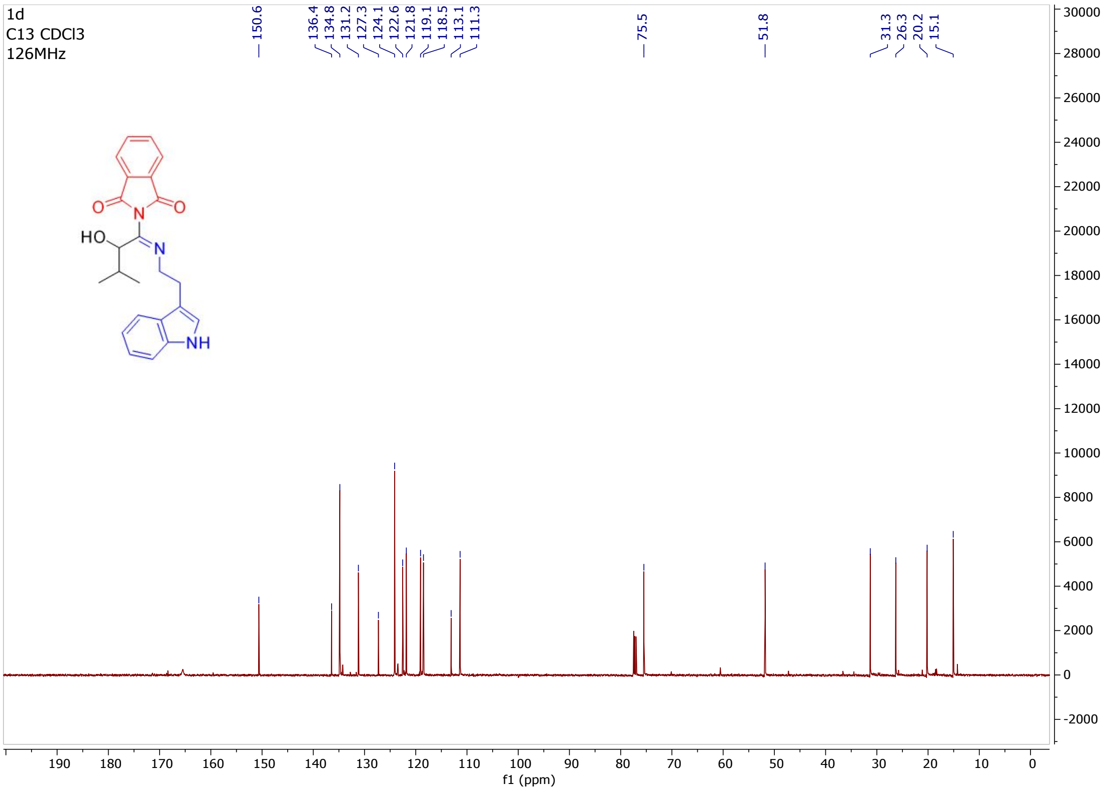

2-(1-(cyclohexylimino)-2-hydroxy-3-methylbutyl)isoindoline-1,3-dione (1e)

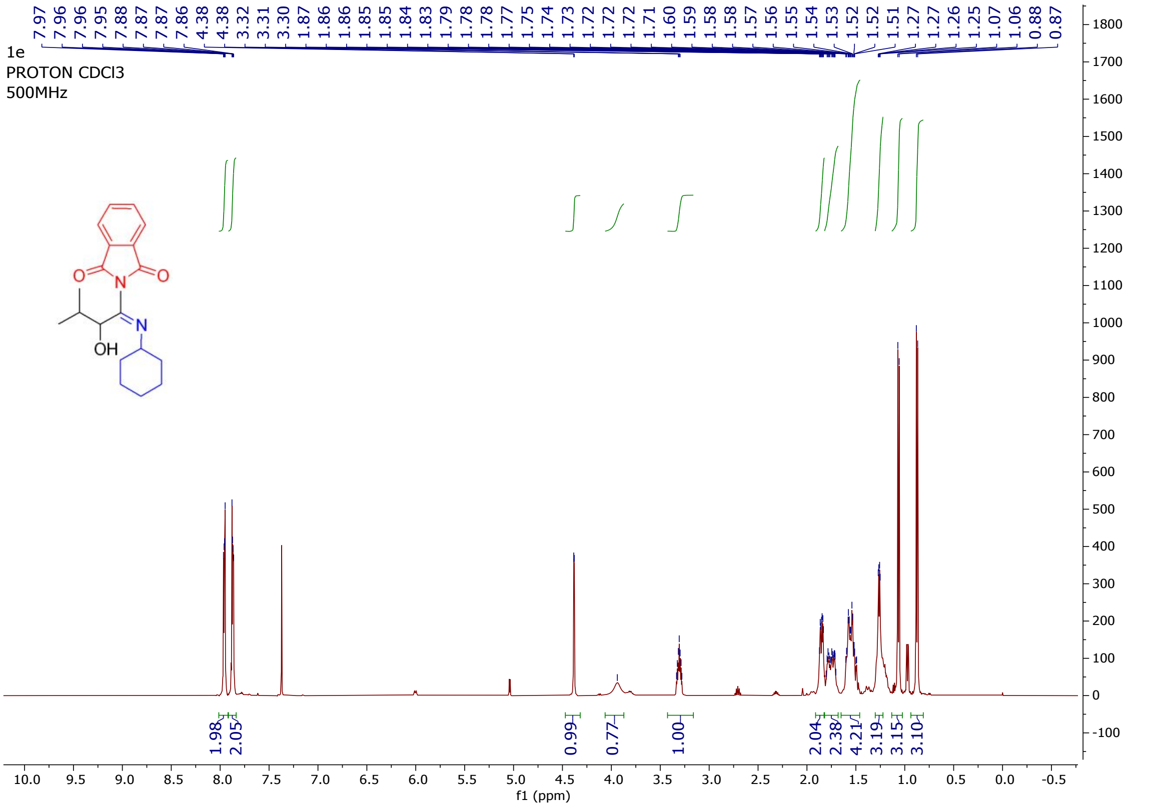

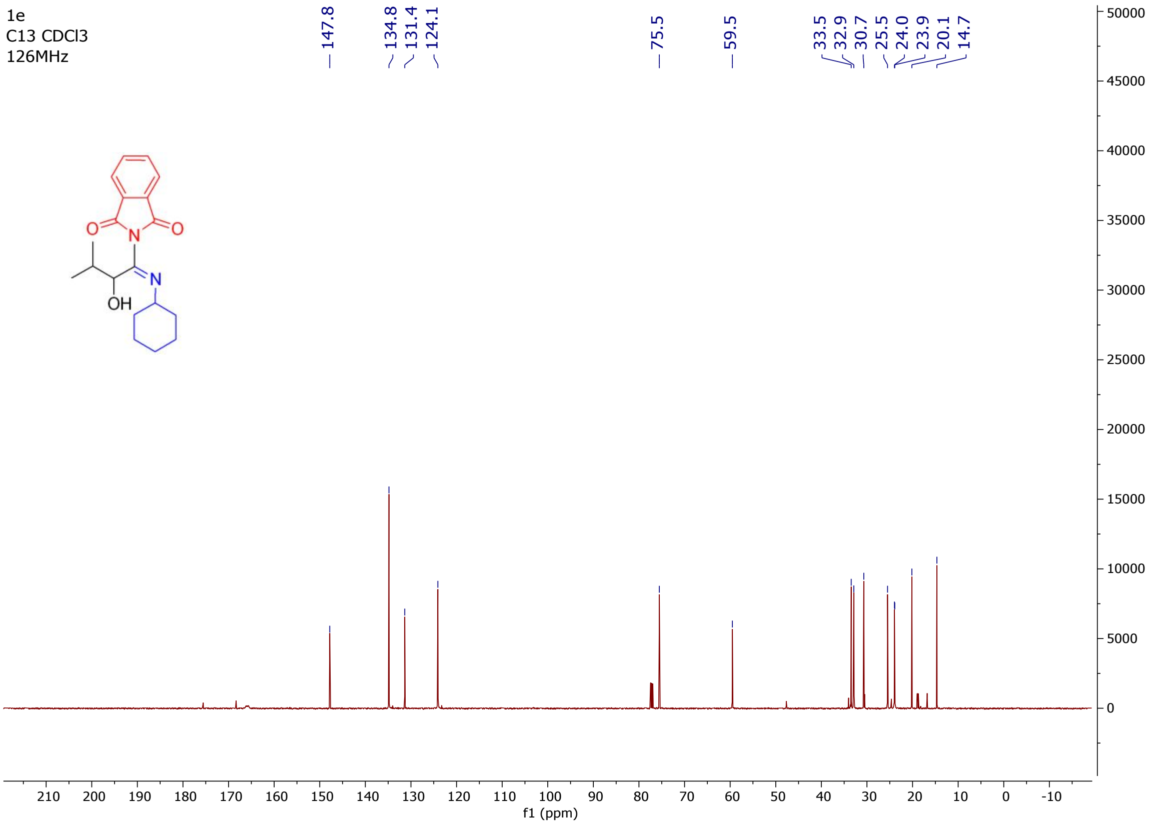

2-((benzylimino)(1-hydroxycyclohexyl)methyl)isoindoline-1,3-dione (1f)

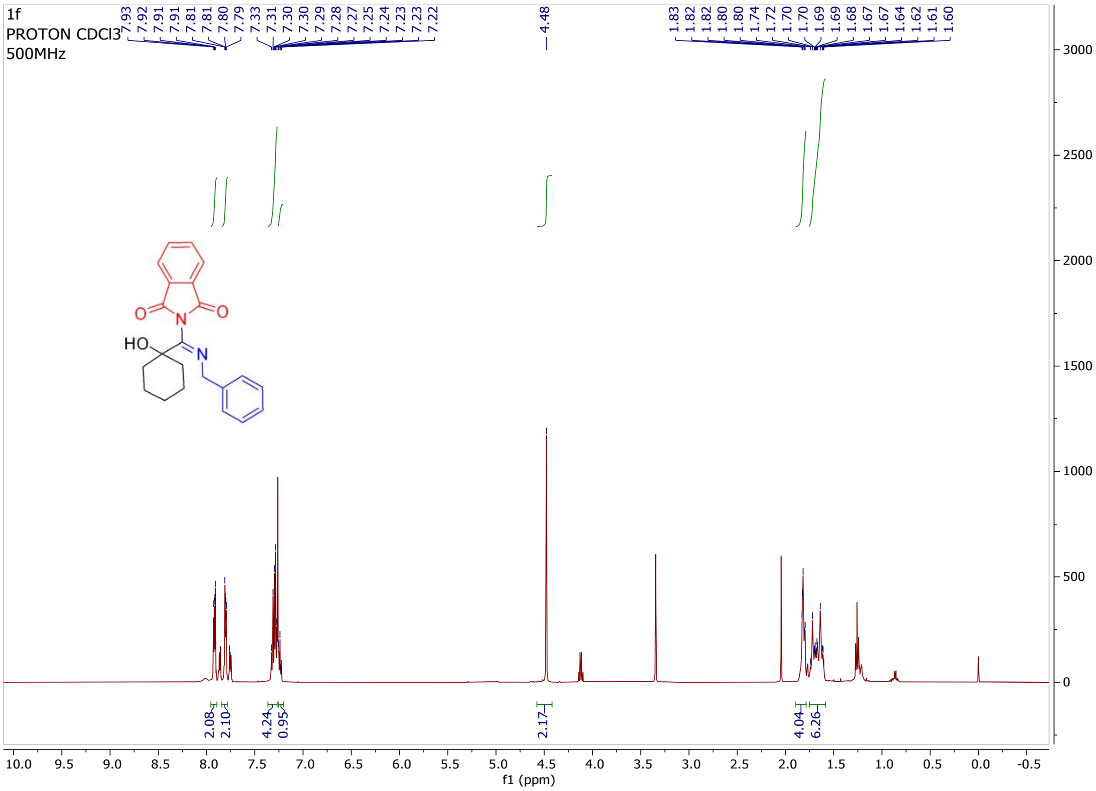

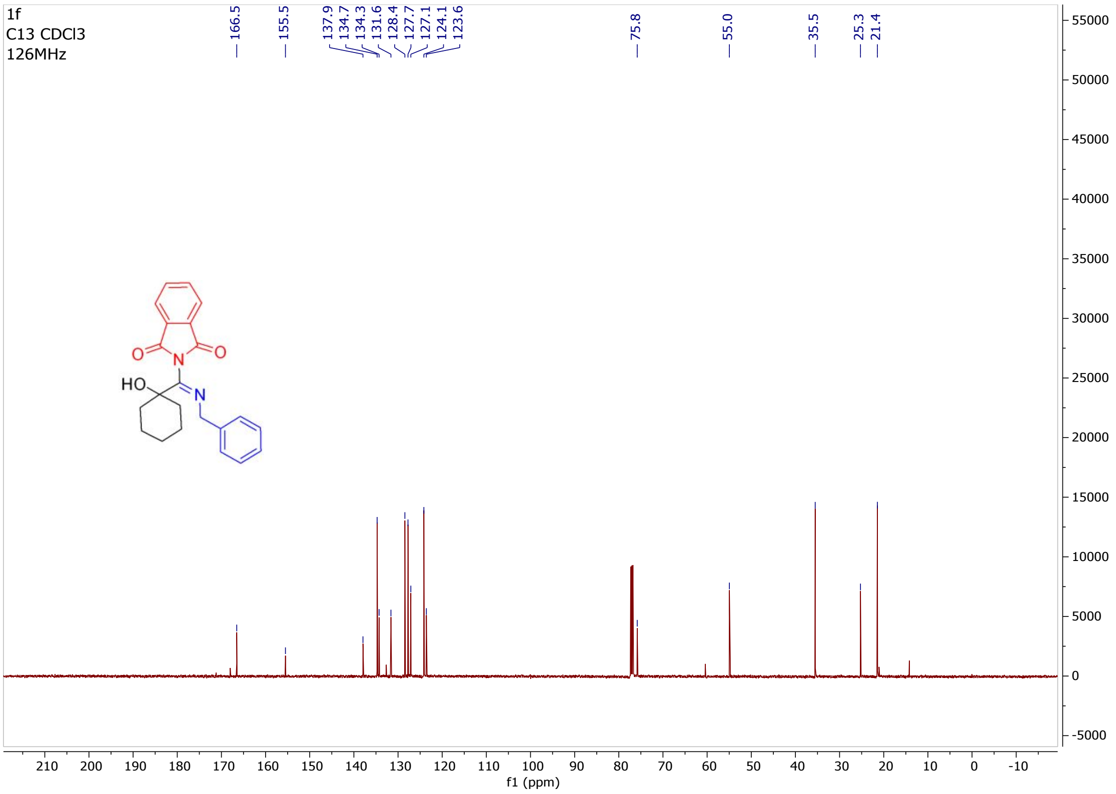

2-(1-(benzylimino)-2-cyclopentyl-2-hydroxyethyl)isoindoline-1,3-dione (1g)

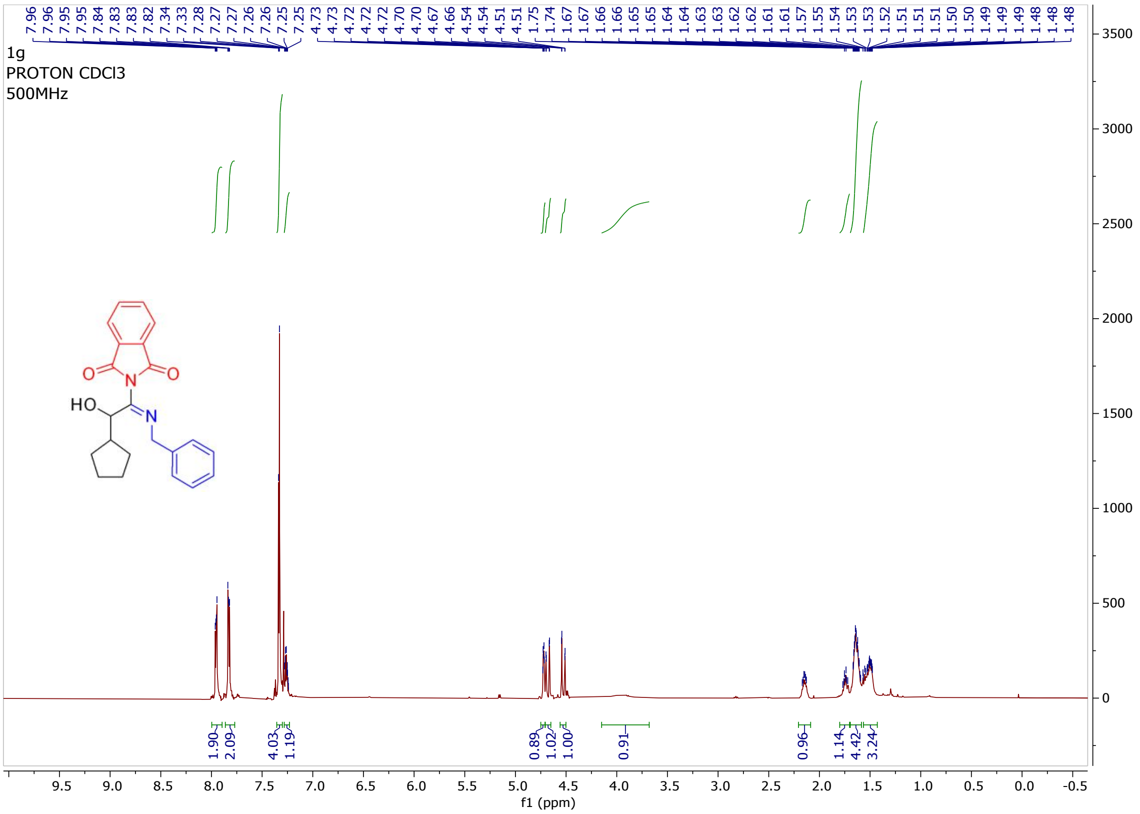

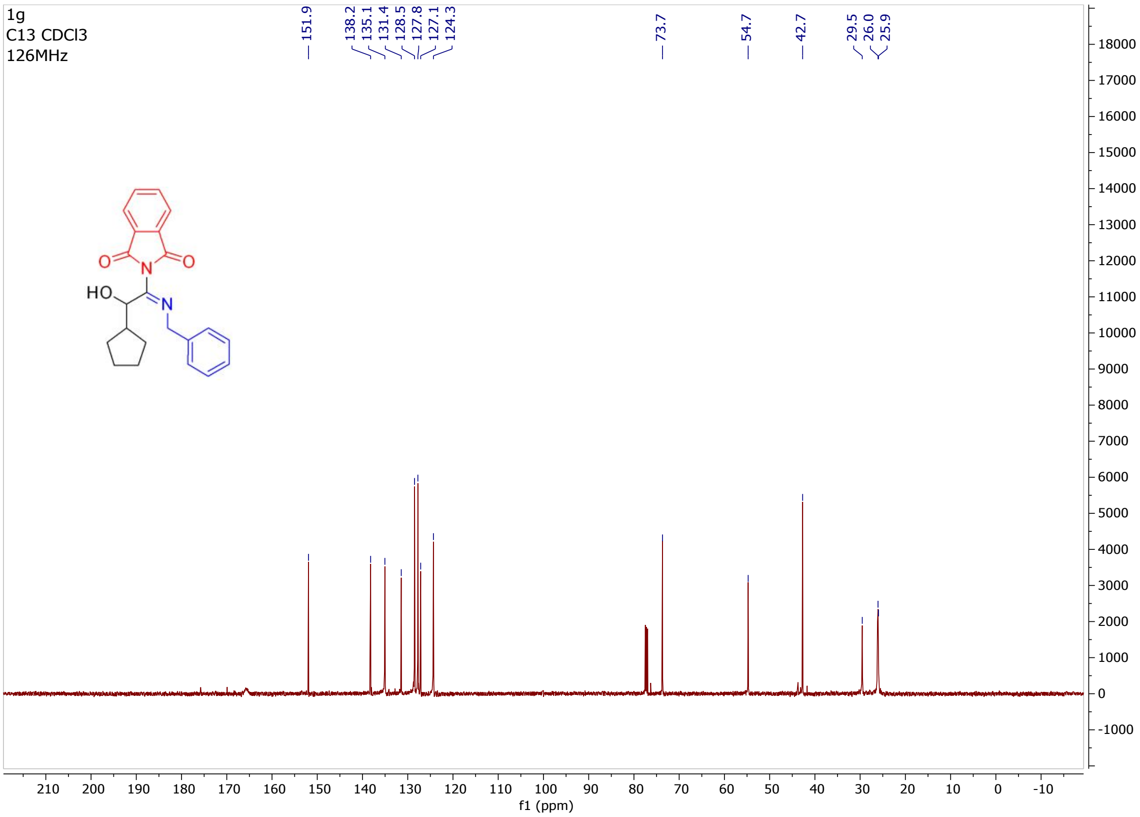

2-(1-(benzylimino)-2-hydroxy-4-methylpentyl)isoindoline-1,3-dione (1h)

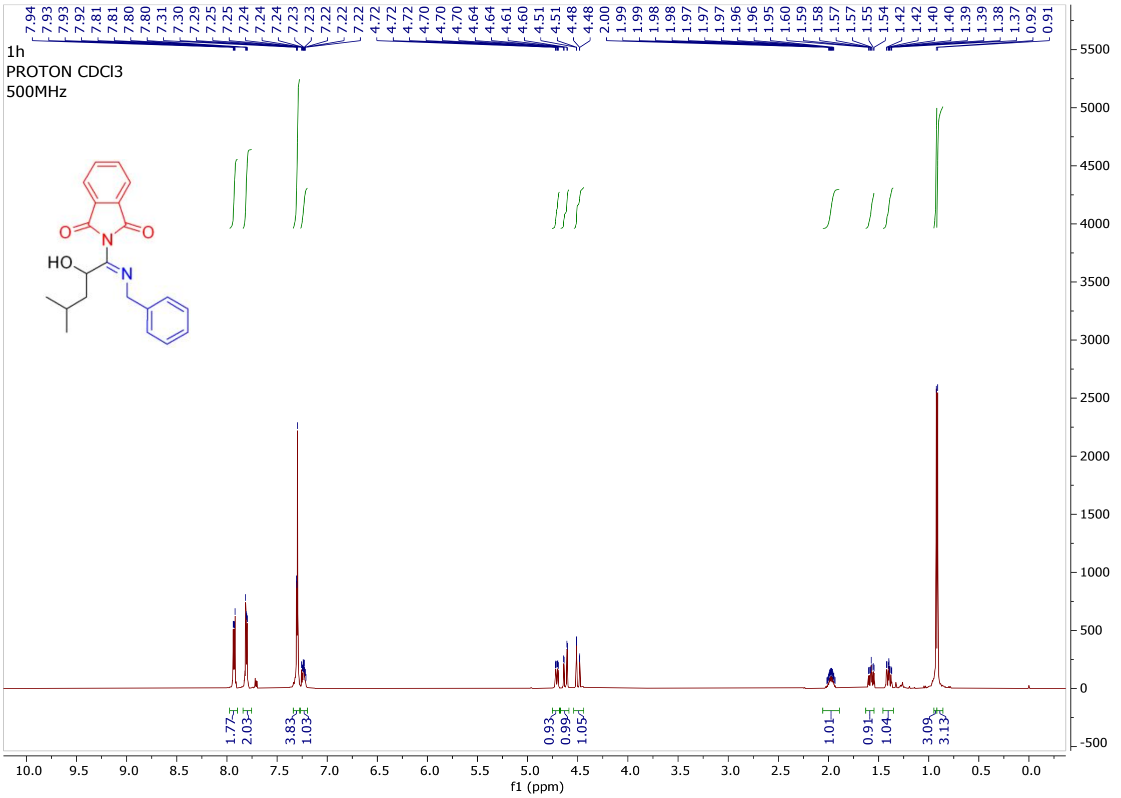

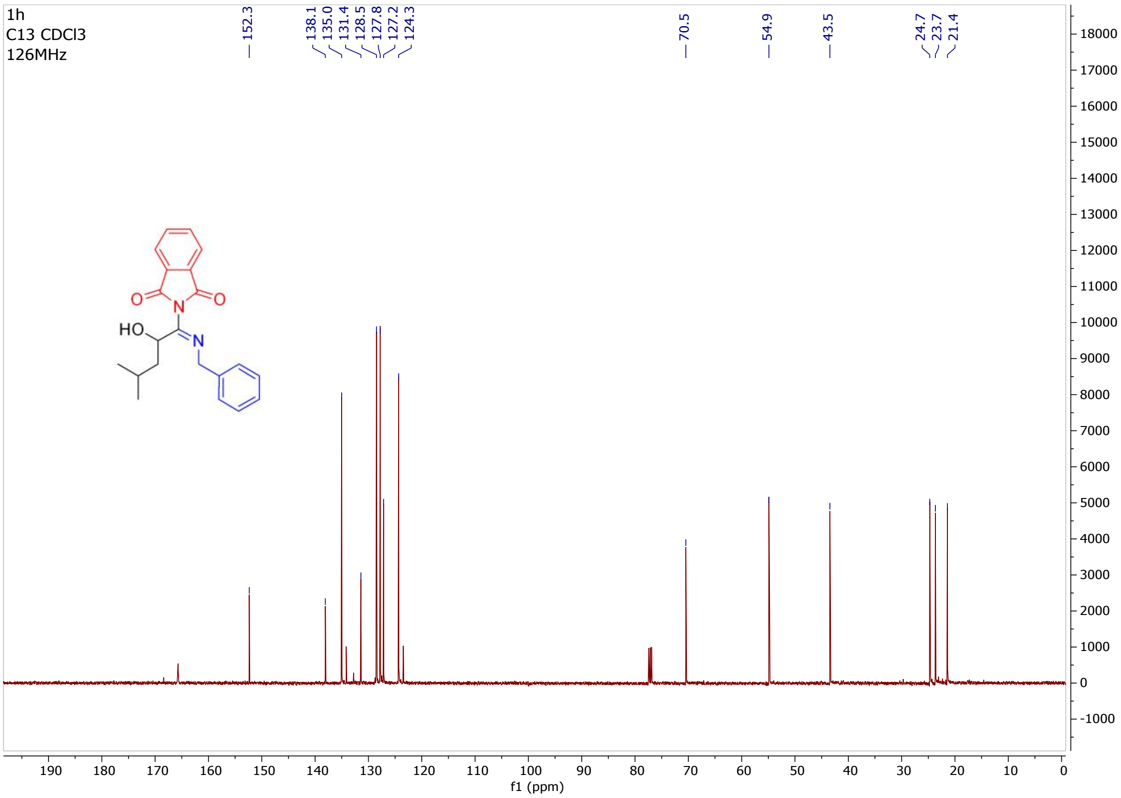

1-(1-(benzylimino)-2-hydroxy-3-methylbutyl)-1*H*-pyrrole-2,5-dione (2a)

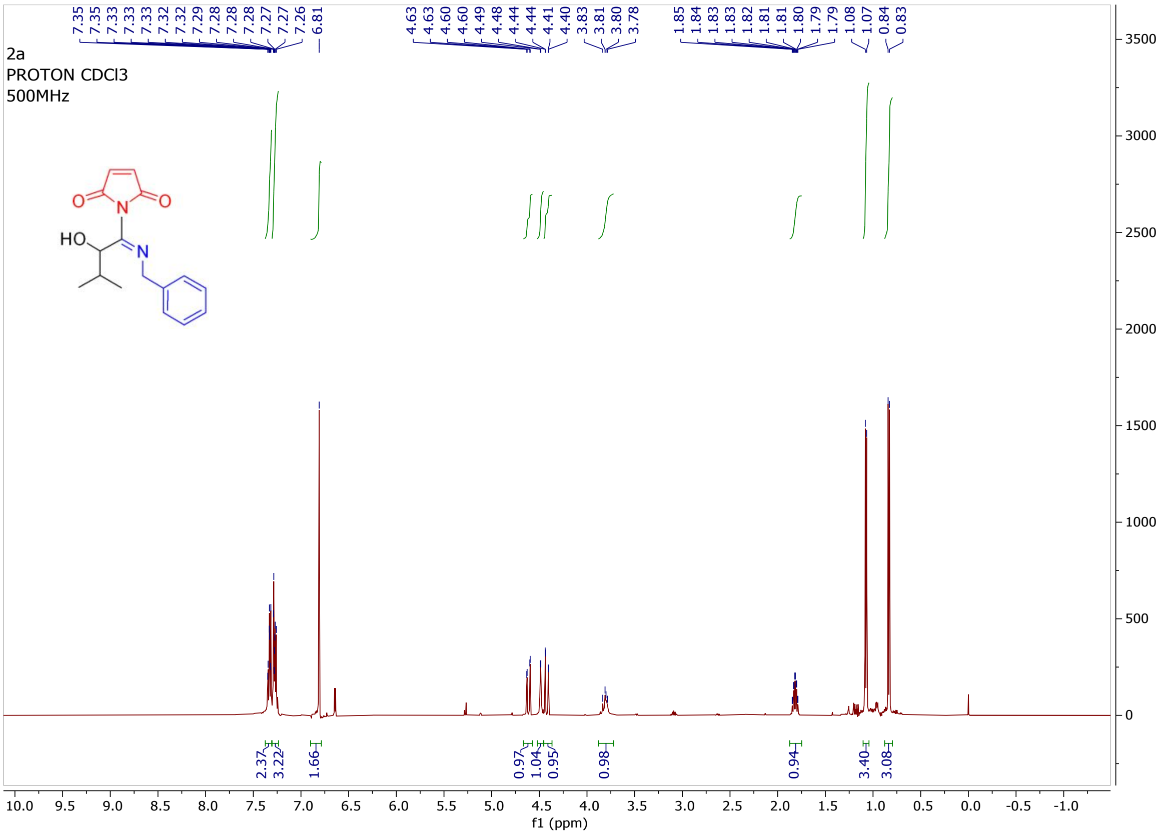

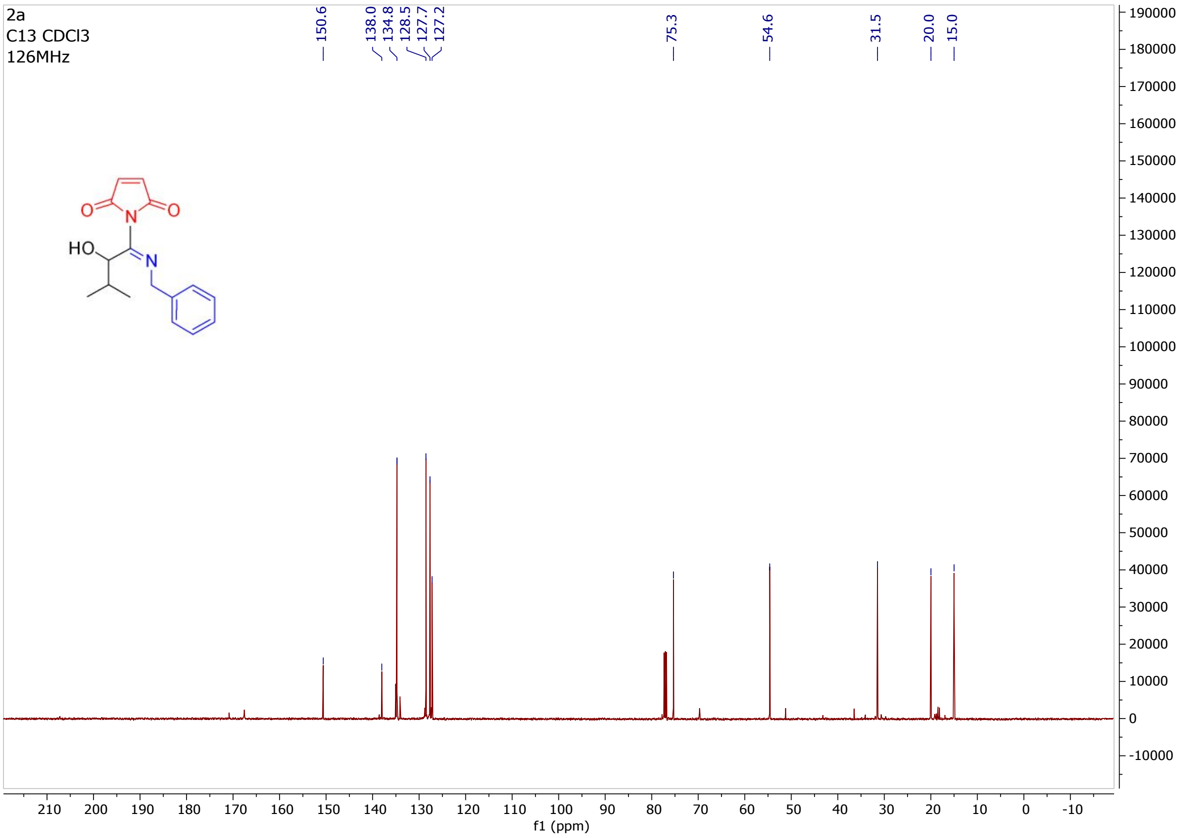

2-(1-(benzylimino)-2-hydroxy-3-methylbutyl)-1*H*-benzo[f]isoindole-1,3(2*H*)-dione (2b)

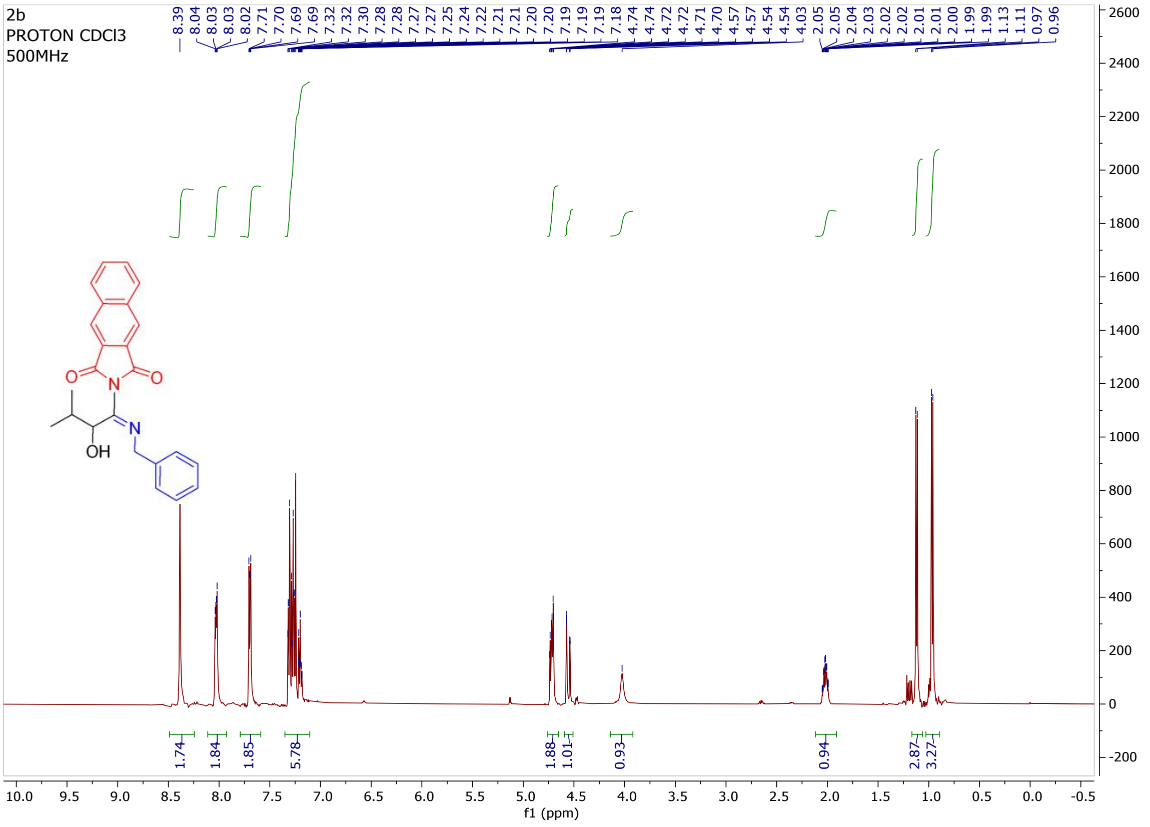

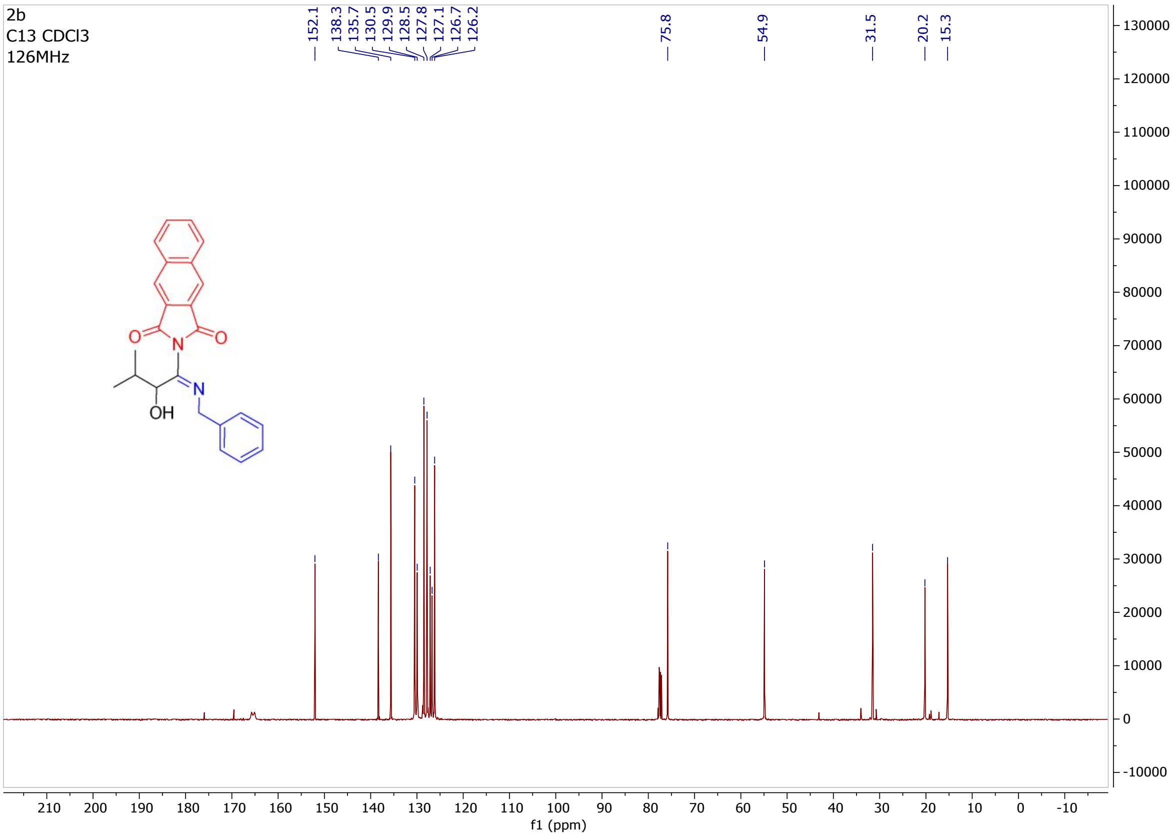

2-(1-(benzylimino)-2-hydroxy-3-methylbutyl)-1*H*-benzo[de]isoquinoline-1,3(2*H*)-dione (2c)

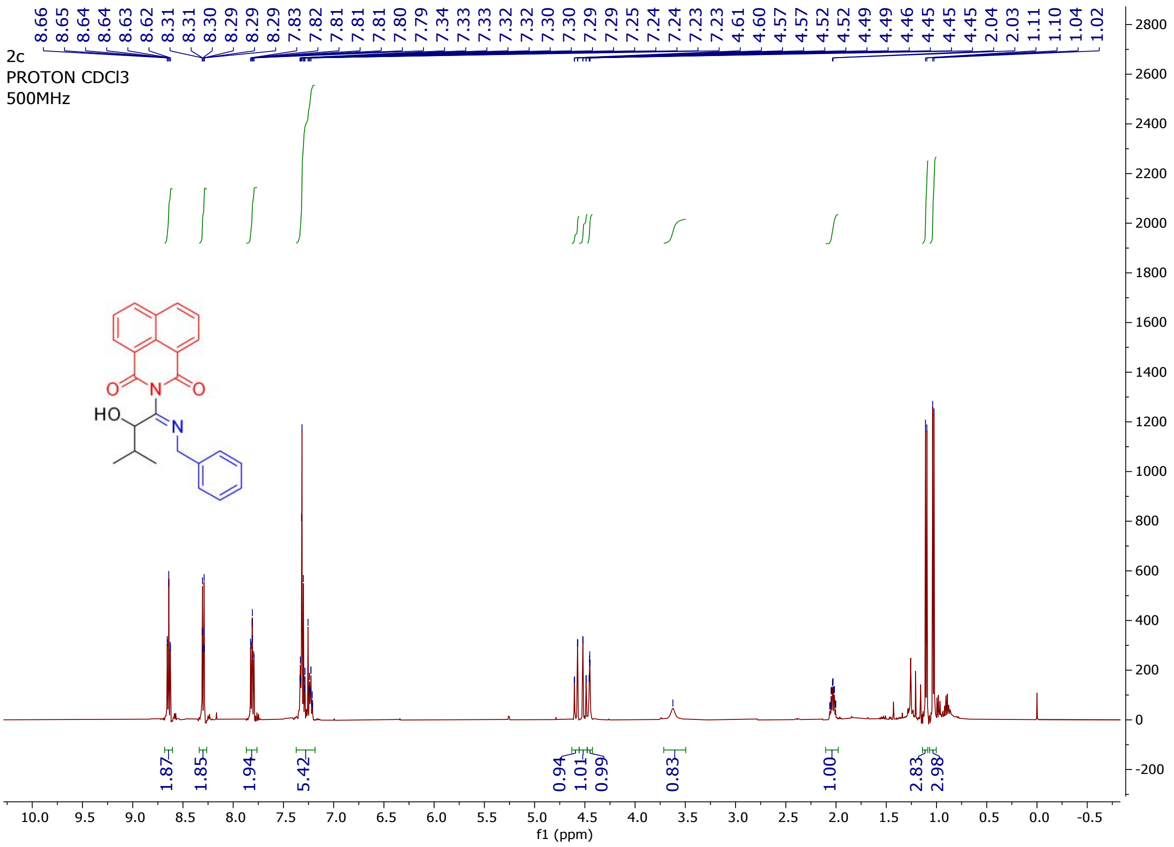

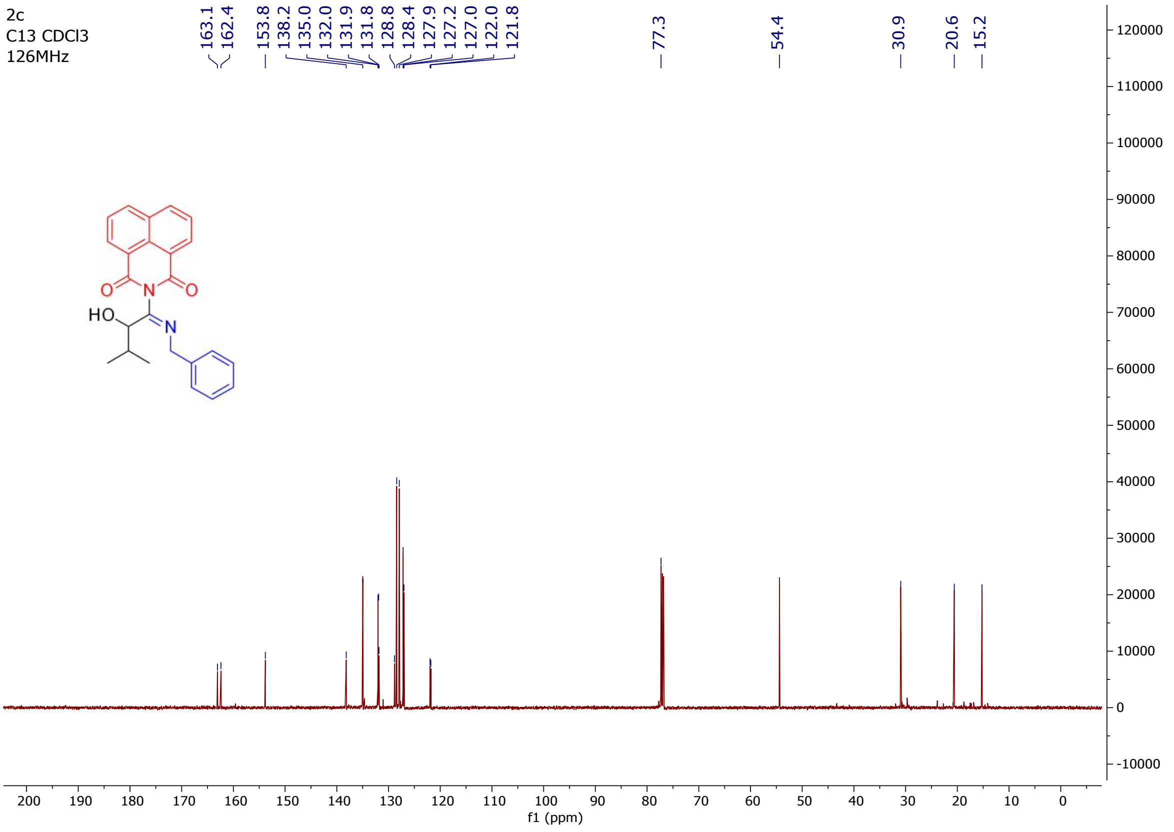

2-(1-(benzylimino)-2-hydroxy-3-methylbutyl)-5-chloroisoindoline-1,3-dione (2d)

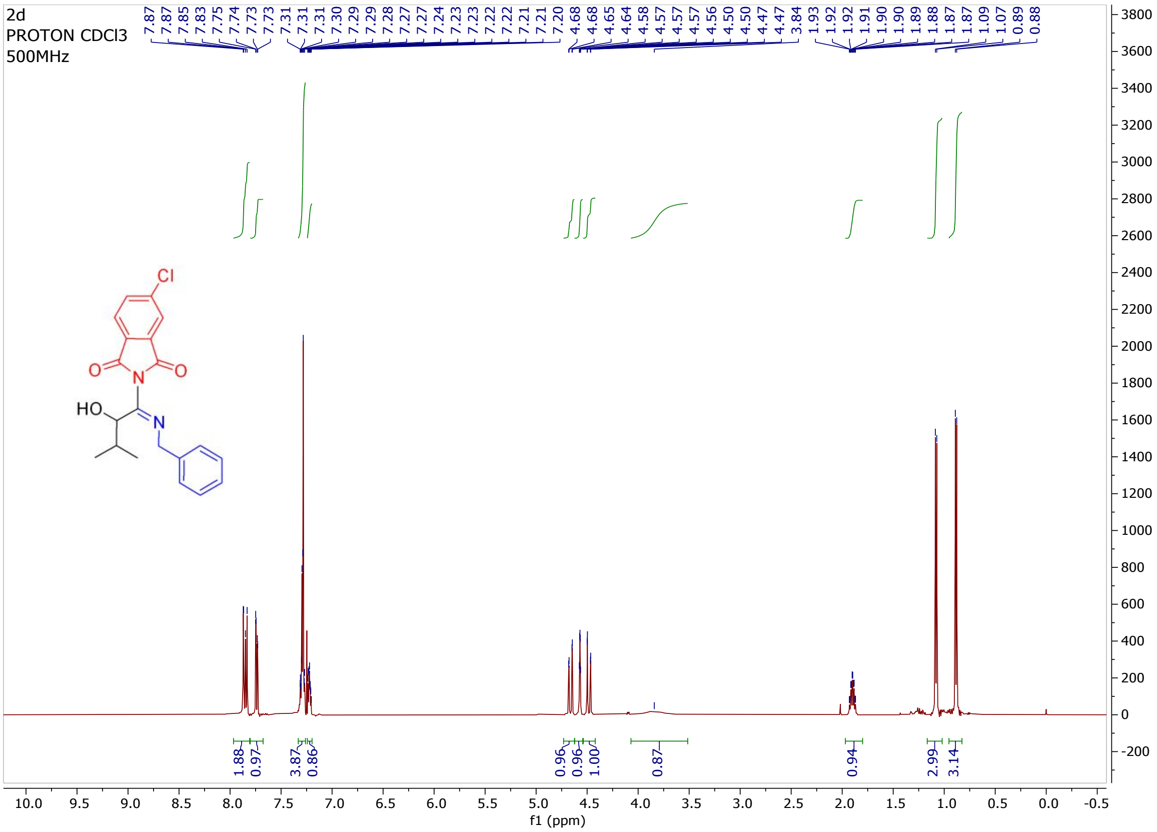

2-(1-(benzylimino)-2-hydroxy-3-methylbutyl)-4-nitroisoindoline-1,3-dione (2e)

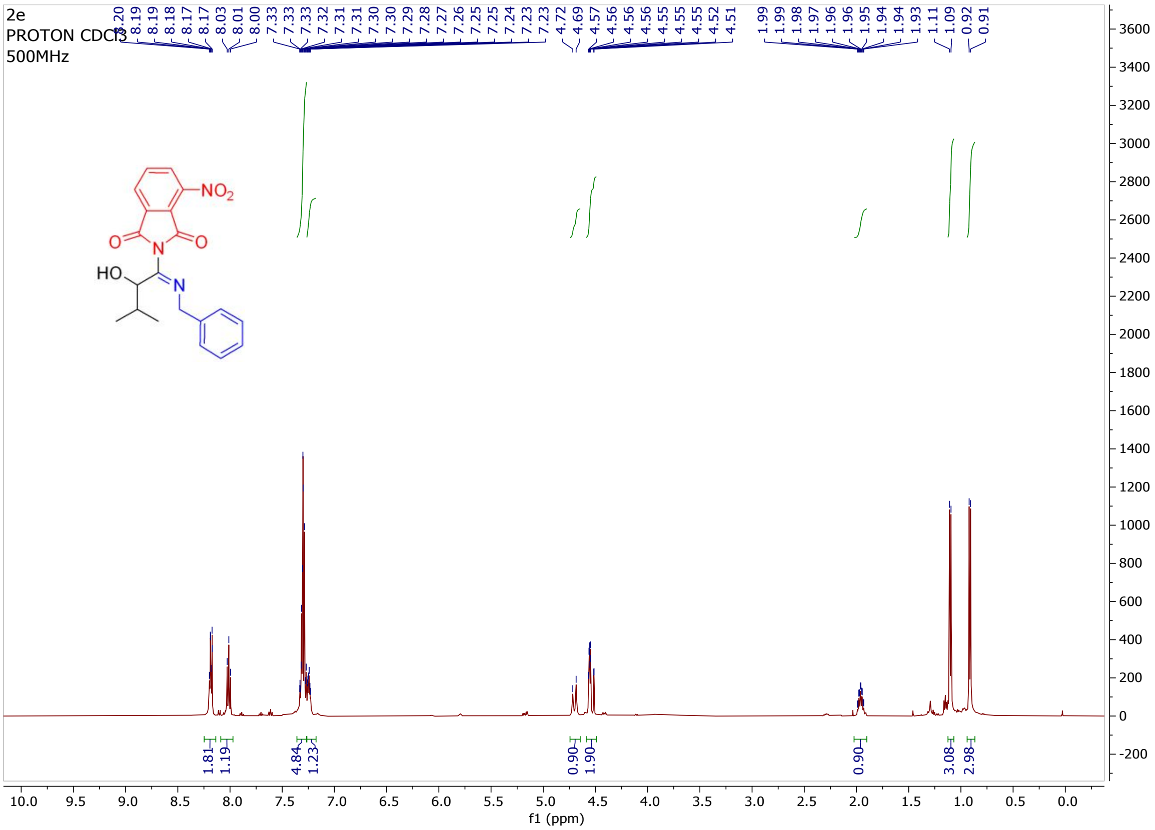

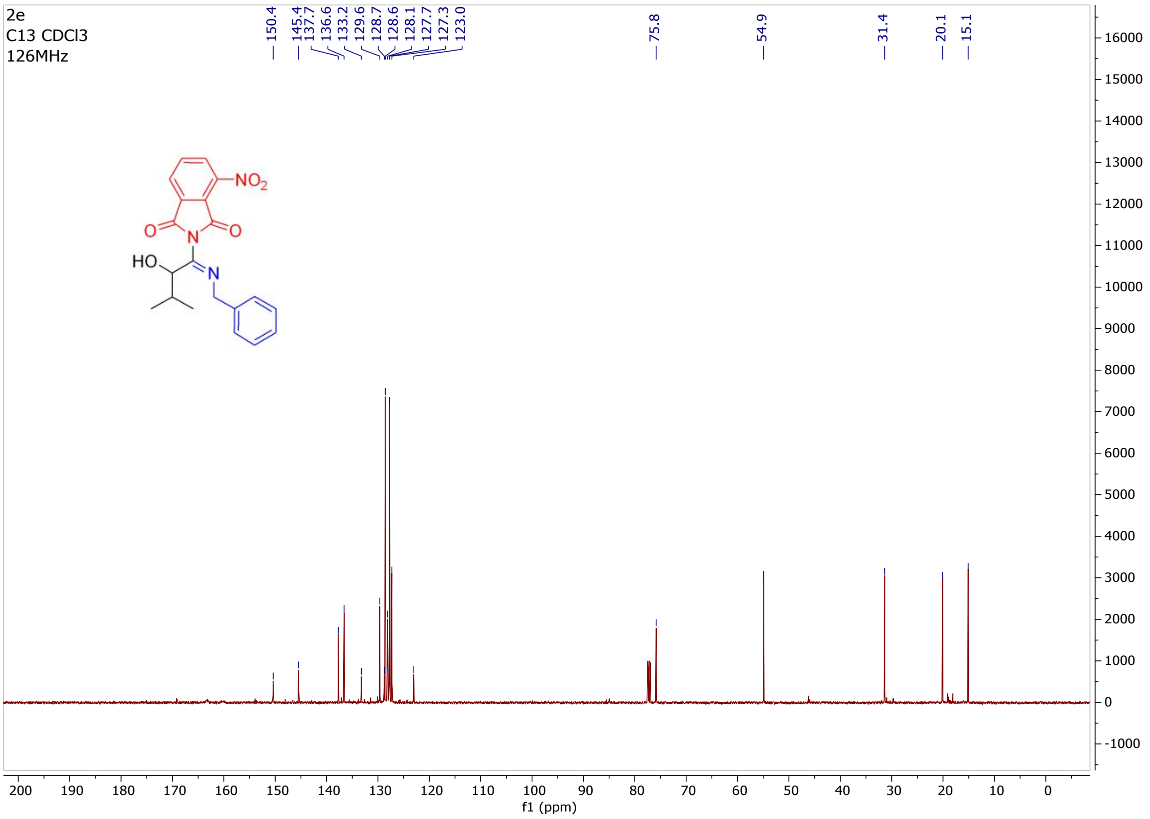

2-(1-(benzylimino)-2-hydroxy-3-methylbutyl)-5-methylisoindoline-1,3-dione (2f)

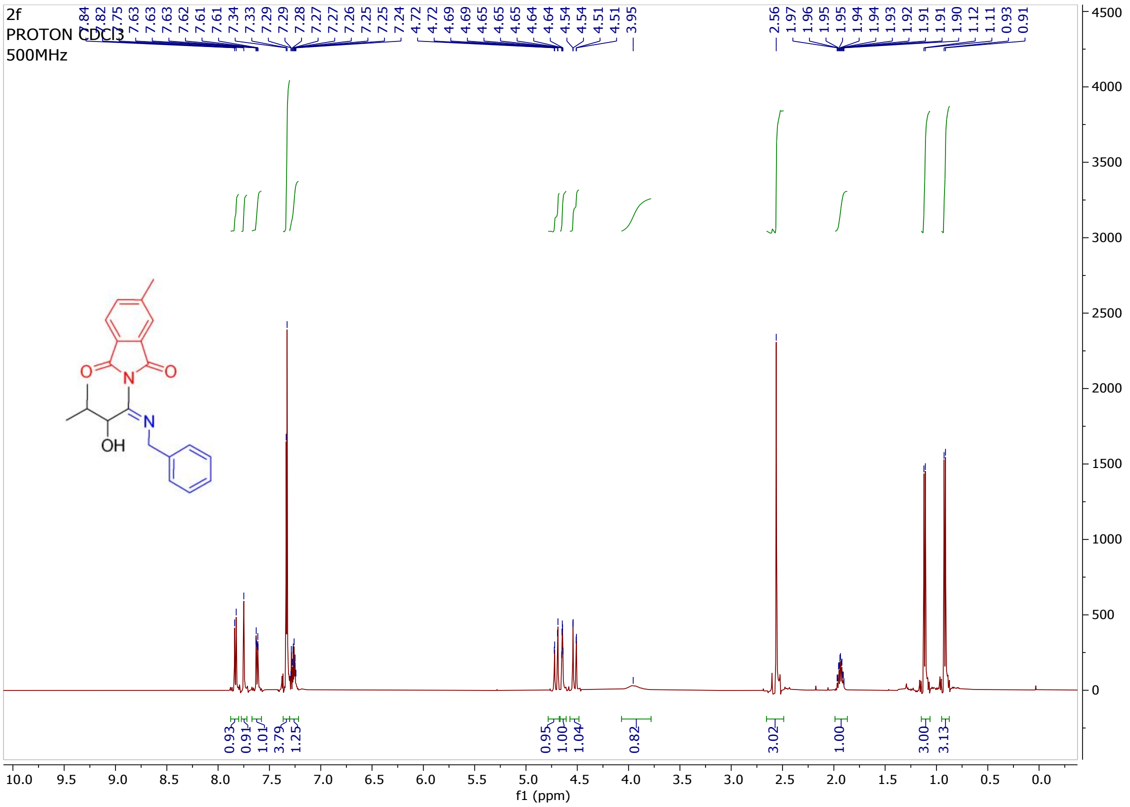

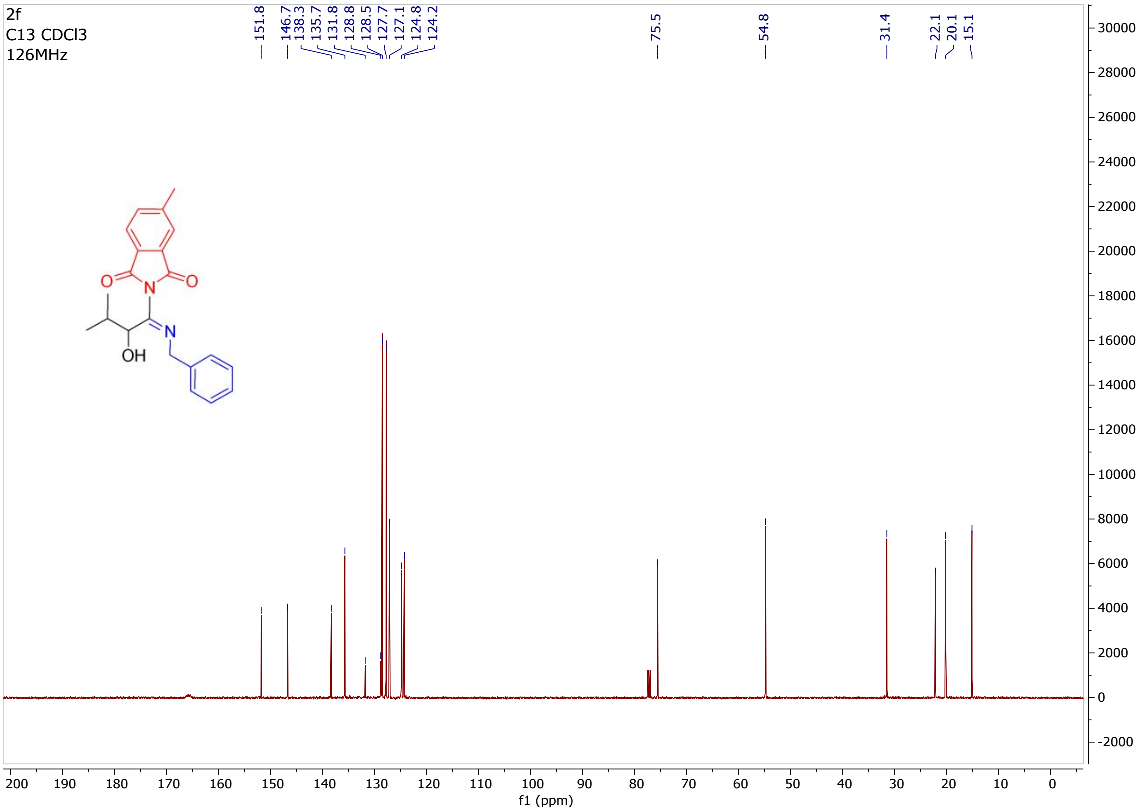

2-(1-(benzylimino)-2-hydroxy-3-methylbutyl)-5-methoxyisoindoline-1,3-dione (2g)

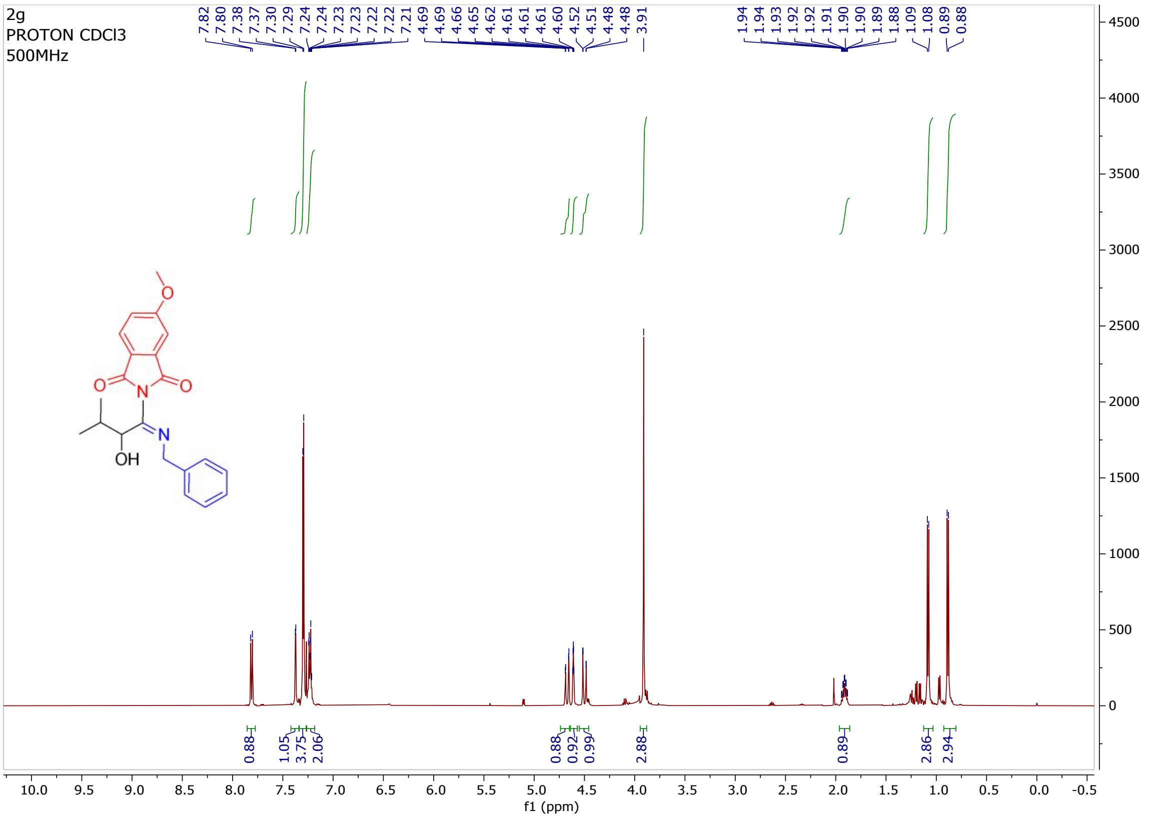

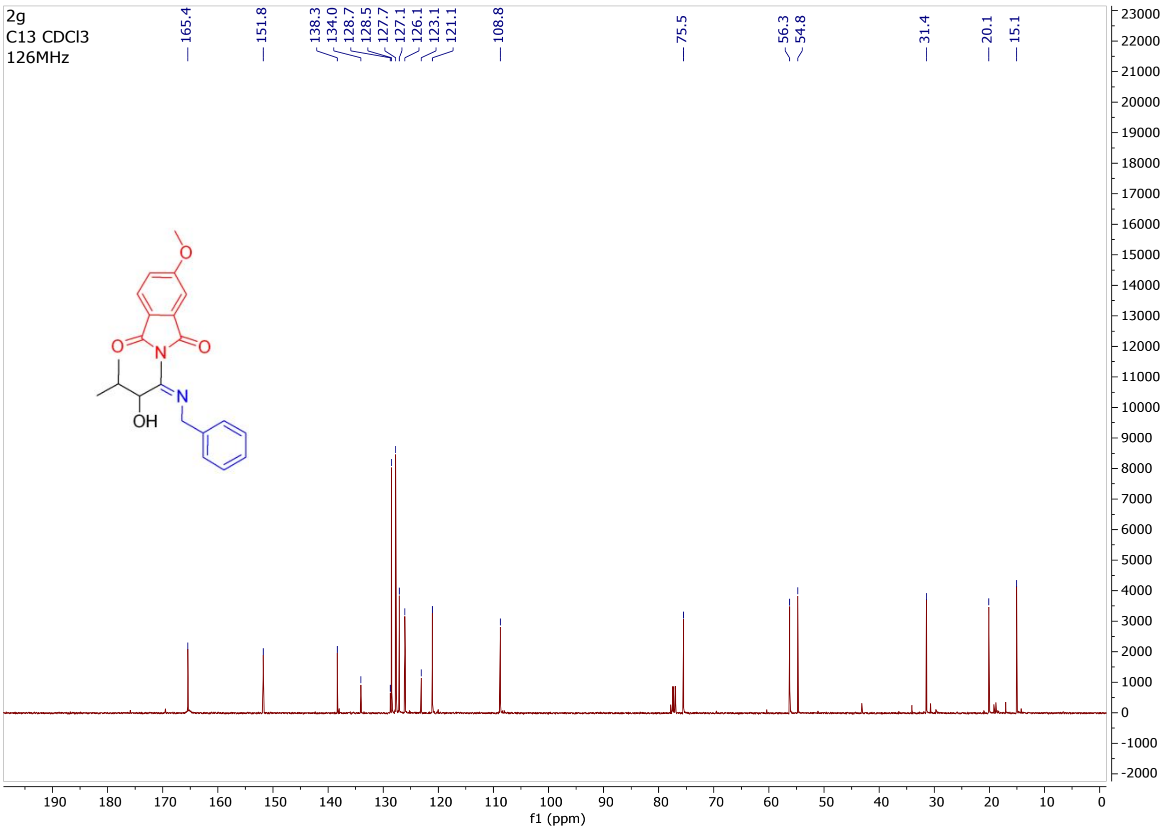

(*Z*)-*N*'-(2-(1*H*-indol-3-yl)ethyl)-2-hydroxy-3-methylbutanimidamide(3a)

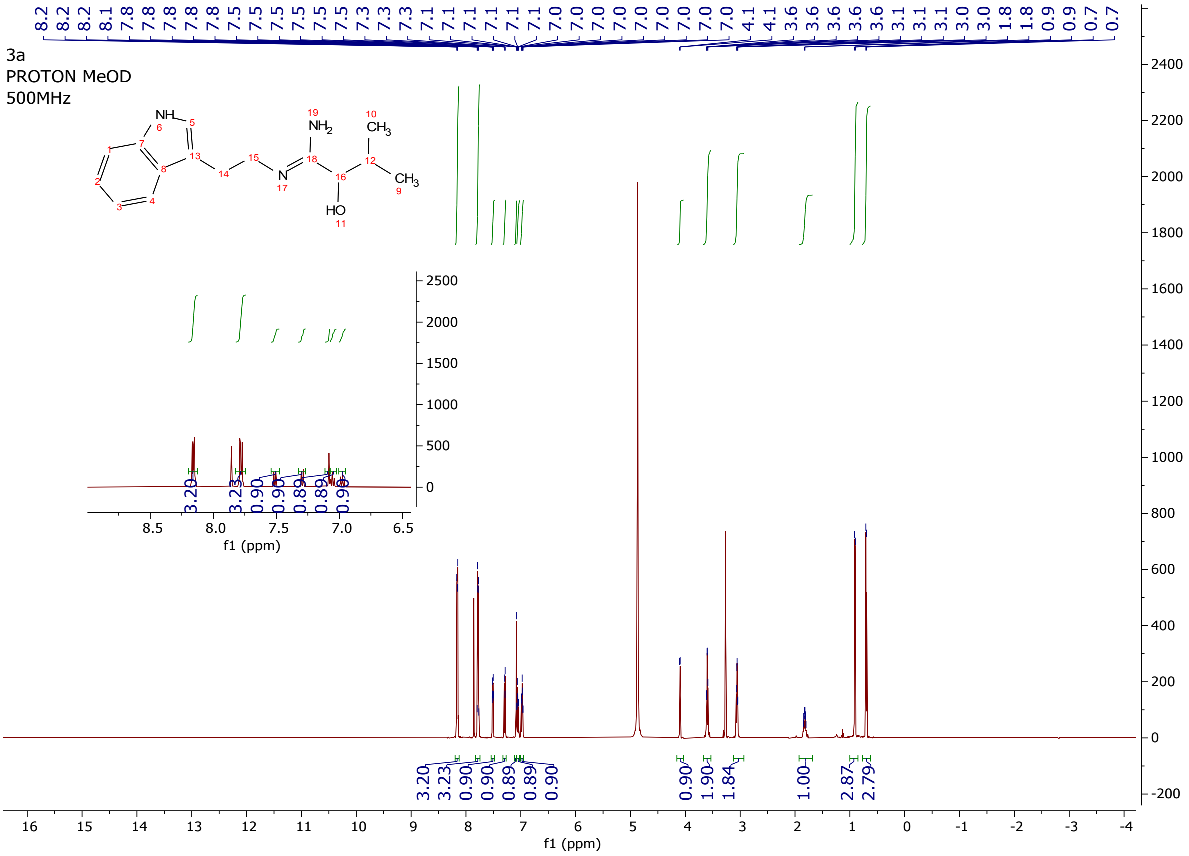

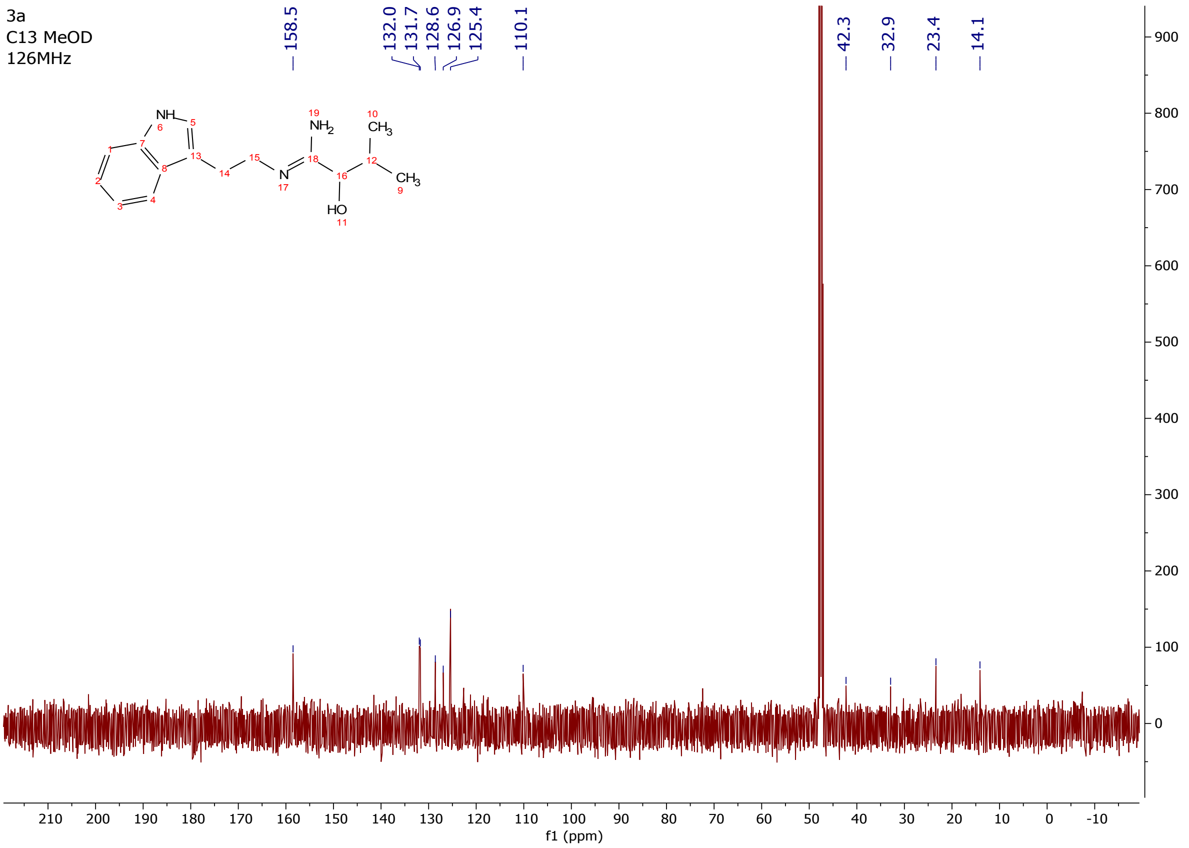

***N*-(2-(1*H*-indol-3-yl)ethyl)-2-hydroxy-3-methylbutanamide(4a)**

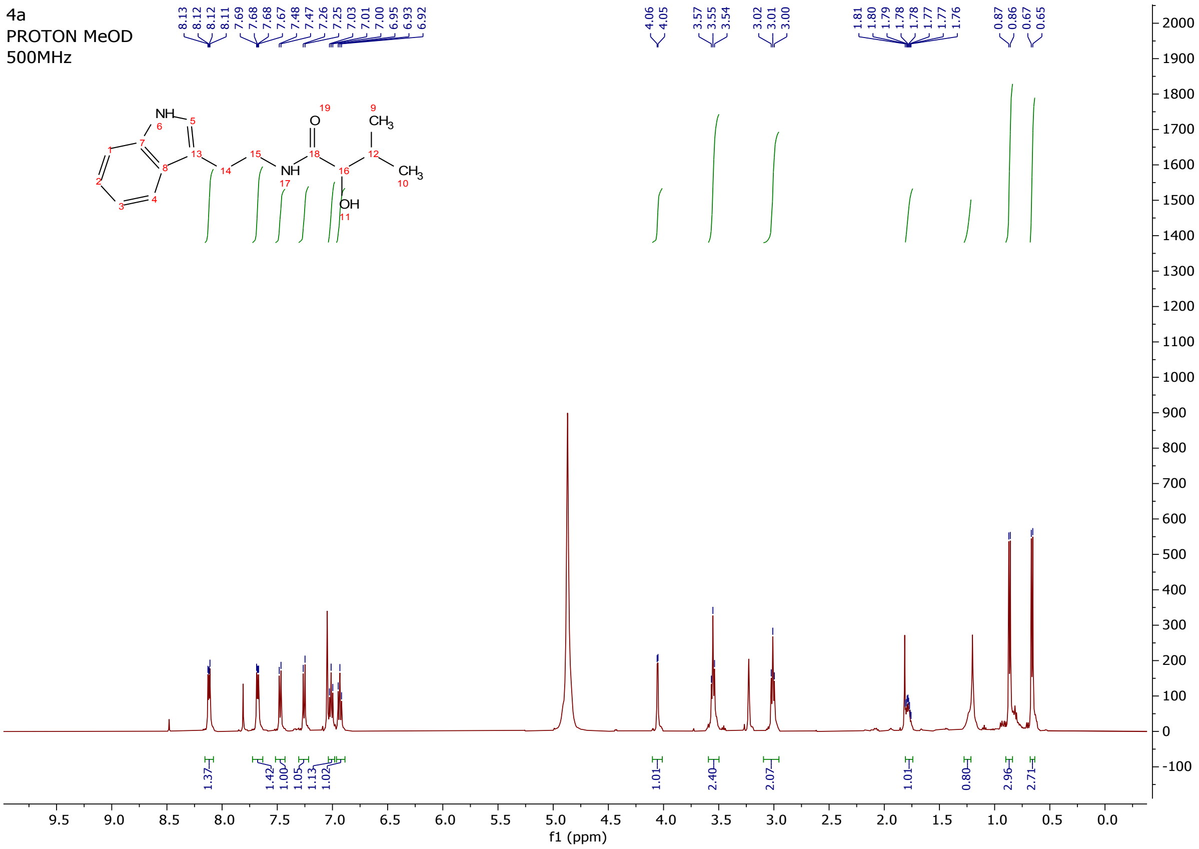
4a
PROTON MeOD
500MHz
f1 (ppm)

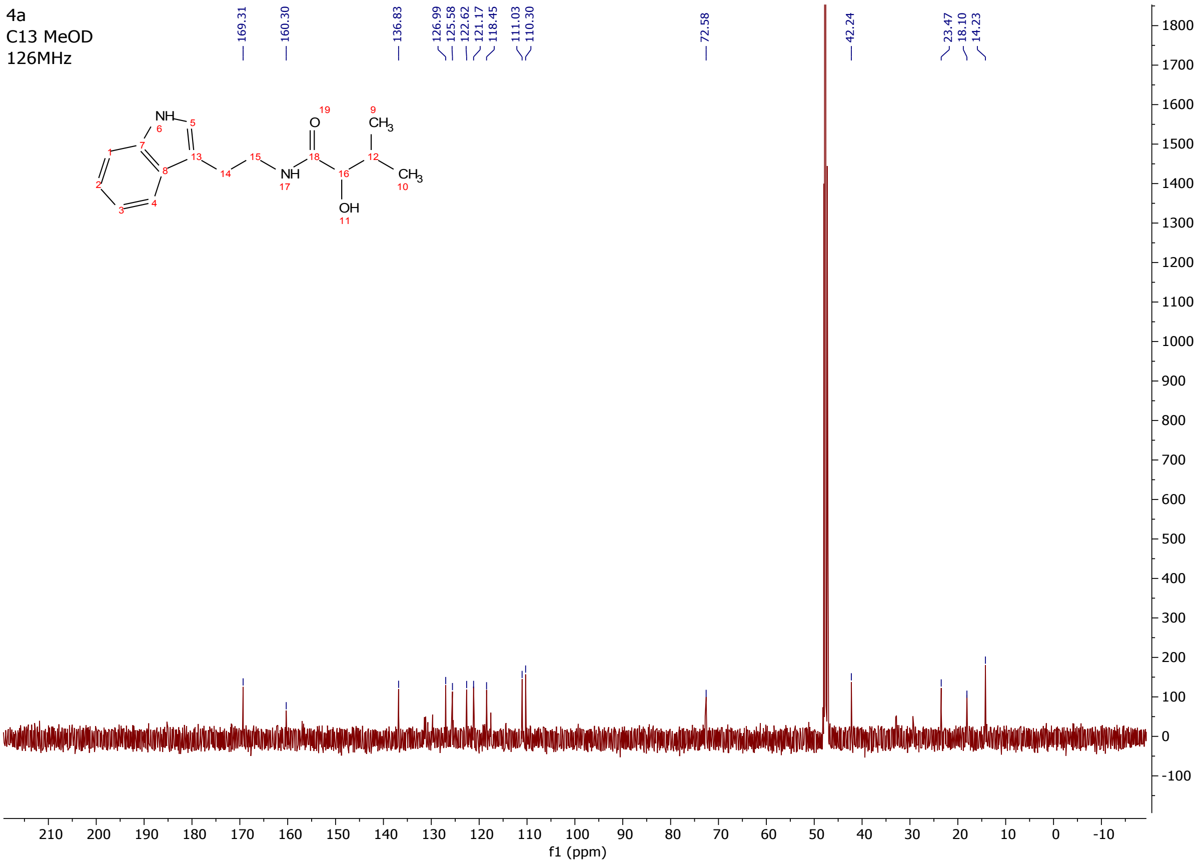
4a
C13 MeOD
126MHz
f1 (ppm)
